# A Rapid Process for Identifying and Prioritizing Technology-Based Tools for Health System Implementation

**DOI:** 10.2196/11195

**Published:** 2018-11-27

**Authors:** Emily R Dibble, Bradley E Iott, Allen J Flynn, Darren P King, Mark P MacEachern, Charles P Friedman, Tanner J Caverly

**Affiliations:** 1 Department of Learning Health Sciences Medical School University of Michigan Ann Arbor, MI United States; 2 School of Information University of Michigan Ann Arbor, MI United States; 3 Department of Health Management and Policy School of Public Health University of Michigan Ann Arbor, MI United States; 4 Division of Hematology and Oncology Department of Internal Medicine University of Michigan Ann Arbor, MI United States; 5 Taubman Health Sciences Library University of Michigan Ann Arbor, MI United States; 6 Ann Arbor Veterans Affairs Center for Clinical Management Research Ann Arbor, MI United States; 7 Department of Internal Medicine Medical School University of Michigan Ann Arbor, MI United States

**Keywords:** patient reported outcome measures, evidence-based practice, decision support systems, clinical, medical informatics applications, practice guidelines as topic, evidence review, expert panel, health information technology, oncology care model, clinical decision support

## Abstract

**Background:**

Health system decisions to put new technologies into clinical practice require a rapid and trustworthy decision-making process informed by best evidence.

**Objective:**

This study aimed to present a rapid evidence review process that can be used to inform health system leaders and clinicians seeking to implement new technology tools to improve patient-clinician decision making and patient-oriented outcomes.

**Methods:**

The rapid evidence review process we pioneered involved 5 sequential subprocesses: (1) environmental scan, (2) expert panel recruitment, (3) host evidence review panel, (4) analysis, and (5) local validation panel. We conducted an environmental scan of health information technology (IT) literature to identify relevant digital tools in oncology care. We synthesized the recent literature using current evidence review methods, creating visual summaries for use by a national panel of experts. Panelists were taken through a 6-hour modified Delphi process to prioritize tools for implementation. Findings from the rapid evidence review panel were taken to a local validation panel for further rapid review during a 3-hour session.

**Results:**

Our rapid evidence review process shows promise for informing decision making by reducing the amount of time and resources needed to identify and prioritize adoption of IT tools. Despite evidence of improved patient outcomes, panelists had substantial concerns about implementing patient-reported outcome tracking tools, voicing concerns about liability, lack of familiarity with new technology, and additional time and workflow changes such tools would require. Instead, clinicians favored technologies that did not require clinician involvement.

**Conclusions:**

Health system leaders can use the rapid evidence review process presented here to usefully inform local technology adoption, implementation, and use in practice.

## Introduction

### Background

Computerized tools that aid patient-provider communication and share medical knowledge are proliferating. Many such tools have also been demonstrated in randomized trials to improve clinical care [1]. These include tools that can support patient self-management (SM) [2], patient decision aids [3], point-of-care clinical decision support [4,5], and Web-based tools that can connect health care teams and patients outside of traditional face-to-face clinic visits, such as tools that automate collection of important patient-reported outcomes (PROs) and feed this information to the clinical care team [6]. These knowledge transfer and communication tools can be broadly categorized as PROs and SM tools. There is high enthusiasm that such tools can help make clinical care more safe, effective, and patient-centered [7].

Despite increasing optimism about the potential for PRO and SM tools to improve clinical care, there are many barriers to their successful implementation [8,9]. These tools can be complex, with multiple components that engage not only patients but also multiple members of the clinical care team [10]. Determining how they best fit into a local health system context is often unclear [11]. Furthermore, the extent to which these tools have been tested varies. Relatively, few have been found effective in clinical practice outside of initial efficacy trials, whose purpose is to consider performance in ideal situations [8,12,13]. At the same time, it is not practical for hospital and health system personnel to spend years formally evaluating these and other systems before implementing them.

### Objective

Health systems and larger clinical communities interested in taking advantage of promising PRO and SM tools need a rapid but still systematic and trustworthy process for identifying, prioritizing, and adapting tools for local implementation [9,14]. Methods of rapid analysis have been developed to aid pragmatic application of research, such as ethnographic style analysis [15,16] and assessment of health technology literature [17,18]. To our knowledge, however, no methods exist to address our question “How can health systems rapidly identify and evaluate technology-based tools that claim to improve clinical care to prioritize them for local use?”

One area where PRO and SM tools have growing policy impetus is oncology care. For example, the Oncology Care Model (OCM) is a pay-for-performance model that emphasizes PRO measures and is being implemented by 192 practices and 14 payers nationwide, including our own academic cancer center [19].

**Figure 1:**
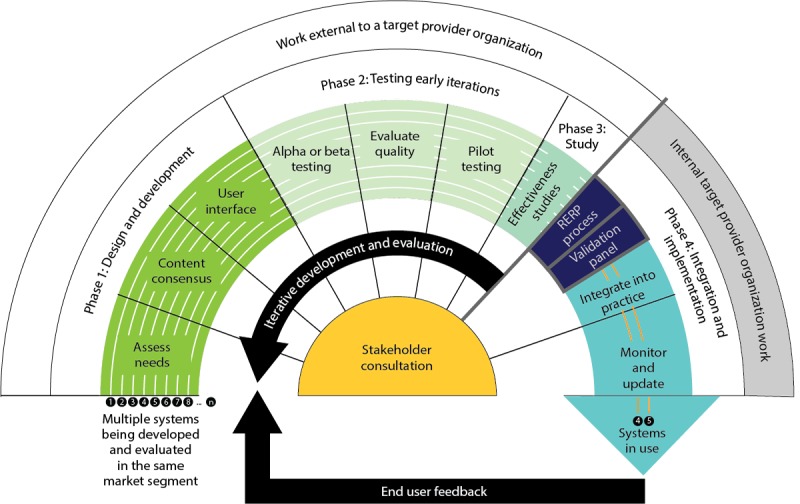
Revised Design and develOpment, Testing early iterations, Testing for effectiveness, Integration, and implementation (DoTTI) framework.RCT: randomized controlled trial.

Thus, OCM provided an ideal test case for developing and evaluating a rapid evidence review process to review PRO and SM tools, with a goal of enabling experts to (1) rapidly evaluate evidence for complex computerized tools and (2) prioritize which tools are put into practice. We called this novel process the rapid evidence review panel (RERP) for PRO and SM tools. This methodology study describes our novel evidence review process and how it worked in the context of prioritizing, for local use, complex computerized tools to improve the patient experience of cancer care. We intend for this to be an efficient process that can rapidly unfold at the organizational level.

The RERP process is just 1 important step in a multistep user-centered framework for developing and implementing new tools. The RERP process is not meant to encompass aspects of tool development or evaluation activities around actual tool implementation. Rather, the focus of RERP process is on rapid and pragmatic evidence review that can be conducted within busy health systems. The goal of the RERP process is to help health system leaders prioritize, using current best evidence, which existing tools may be the most feasible and important to implement. The RERP process is important because, if health systems are able to adopt such a process in a systematic and ongoing fashion, it opens a potential path for important new technologies to be adopted more quickly. The RERP process fits within the larger *Design and develOpment, Testing early iterations, Testing for effectiveness, Integration and implementation* (DoTTI) development and evaluation framework, as portrayed in Figure 1 [20]. The DoTTI framework offers a complete development model for digital tools for patient use. The involvement of patients as stakeholders in the development of PRO and SM tools is essential to ensure that tools meet patient needs and expectations.

## Methods

### Overview

We developed our process to take advantage of existing measures, rapid evidence review methods, consensus-based decision-making methods, and rapid qualitative analysis methods [17,18,21]. The process we developed attempts to streamline the information provided to an expert panel and enable the panel to meet just twice to evaluate and prioritize multiple interventions, once in a 1-hour introductory teleconference and again in a face-to-face 5-hour meeting. This time frame may be adjusted according to the quantity of manuscripts needing to be reviewed.

Given this limited amount of time, it is not practical or efficient for panelists to review full manuscripts, fully review the literature, or individually evaluate evidence. Instead, the RERP process makes use of established evidence review tools and frameworks to ensure a rapid process that is also credible. By shifting the labor to a smaller project team that can collect and synthesize relevant information in advance of expert panel review, the expert panel’s evidence review can be accelerated. Our project team consisted of an oncology subject matter expert, an evidence-based medicine expert, a project manager, and a research specialist. Our project team required approximately 3 months to assemble the evidence presented in the RERP meeting.

**Figure 2:**
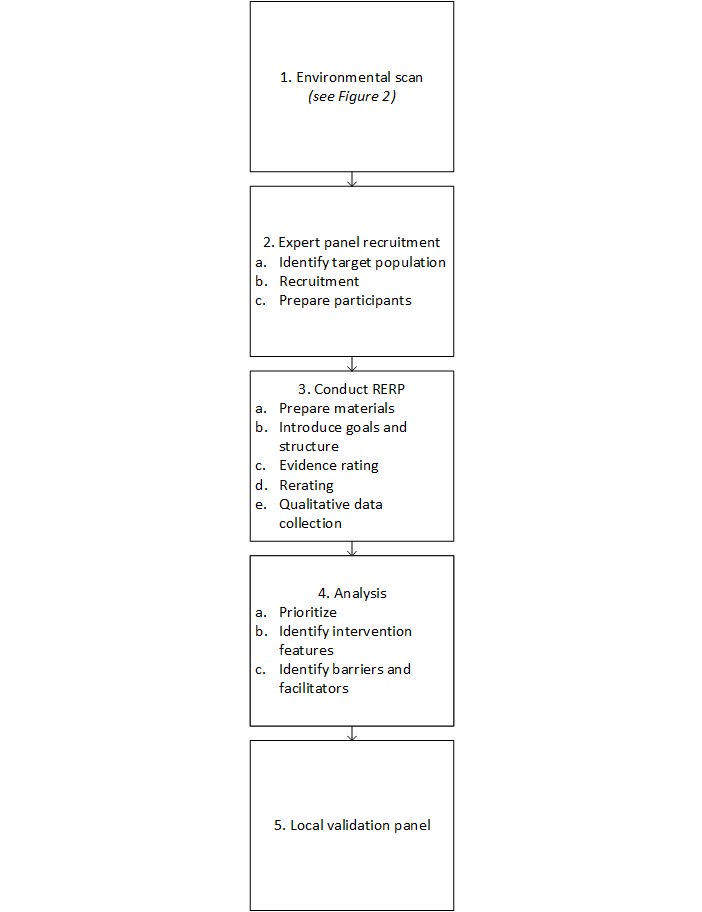
Rapid evidence review panel (RERP) process. This diagram shows each step of the RERP process.

We then used a rapid template-based coding method using the tailored implementation for chronic disease (TICD) framework and developed a categorization scheme for interventions to rapidly interpret the expert evaluations from the RERP [22]. Our aim was to use these findings to inform local effectiveness, implementation, or hybrid studies [23]. We describe each step of the RERP process in detail (Figure 2) below.

### Step 1: Conduct a Rapid Environmental Scan to Identify Promising Tools

**Figure 3:**
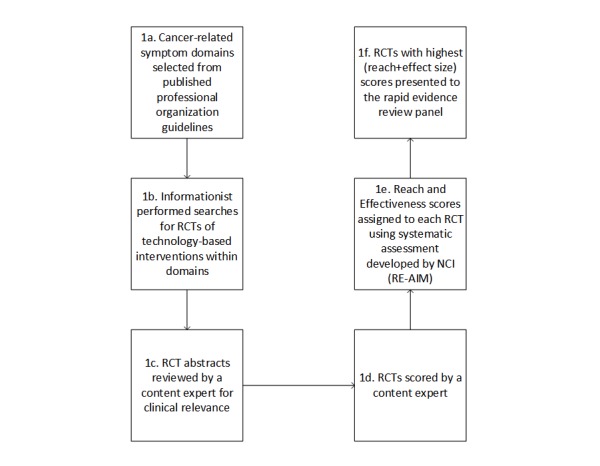
Environmental scan showing the components of Step 1 of the rapid evidence review panel, the environmental scan. RCT: randomized controlled trial; NCI: National Cancer Institute; RE-AIM: reach, effectiveness, adoption, implementation, and maintenance.

A number of procedures exist to conduct rapid reviews of scientific literature [17,18,21]. We chose to conduct a thorough environmental scan (Figure 3) [17]. We first sought to identify the relevant topic domains. In the context of OCM’s incentives to improve the patient experience of cancer care, we focused on PRO and SM tools related to improving cancer and cancer treatment–related symptoms. We identified symptom domains by reviewing all published care guidelines from major professional organizations writing guidelines for any aspect of the cancer care continuum (prevention, screening, diagnosis, treatment, and prognosis). Our review included the following organizations: American Society of Clinical Oncology, National Comprehensive Cancer Network, European Organization for Research and Treatment of Cancer, National Cancer Institute, US Preventive Services Task Force, American Academy of Hospice and Palliative Medicine, American Cancer Society, and the Oncology Nursing Society. This review established the set of possible symptom domains for further study (see Multimedia Appendix 1). From this larger set of symptoms, we prioritized those that applied to multiple different cancers treated in a cancer center to be more relevant to a broader group of patients (eg, we included chemotherapy-induced nausea and vomiting, but excluded highly disease-specific symptoms such as lymphedema in breast cancer patients). Such highly disease-specific symptoms are certainly important for consideration but were not the focus of our review.

An informationist then performed a systematic search for PRO and SM tools that targeted one or more of the selected symptom domains and evaluated in randomized controlled trials (RCTs; search strategy described in detail in Multimedia Appendix 2). We chose to focus our search on randomized controlled efficacy trials because an initial search identified few to no implementation or effectiveness studies of PRO and SM tools in these domains. The search strategy we developed to identify PRO and SM tools will be of particular interest to those interested in implementing PRO and SM tools and is described in detail in Multimedia Appendix 2. Moreover, the standardization and internal validity of RCTs aid a rapid and rigorous expert panel evaluation. However, we recognize the need to sometimes move beyond the RCT, particularly in the context of complex interventions such as decision support, where local context and clinical workflows are likely to be key factors in determining the success of the intervention [24-26]. In the absence of large pragmatic trials and implementation studies, single-center and multicenter efficacy studies are likely the best starting points for identifying promising tools.

A content expert on the project team (DK) then reviewed the abstracts of all RCTs retrieved by the search strategy. Our criteria for selecting a local content expert are as follows: (1) clinical expertise in oncology, (2) interest in technology-enabled interventions to improve the patient experience of cancer care, and (3) time and ability to work closely with the evidence-based medicine expert and conduct a thorough review. Although the content expert led the abstract review process, the process also included weekly meetings with an evidence-based medicine expert (TC) to review the abstracts and rationale for inclusion or exclusion. The content expert excluded interventions that were not technology and knowledge based or were not targeting one of the selected symptom domains. Afterwards for further review, he selected those interventions reporting at least some evidence of efficacy in the abstract. Full manuscripts were retrieved for these trials and were read in full by the oncologist. Some manuscripts were excluded at this stage because of the limited clinical relevance of the findings. The oncologist then assigned *effect size* and *reach* scores to each RCT based on a process developed by the National Cancer Institute (ie, using the Research-Tested Intervention Programs review process) [27]. Those RCTs with combined scores (effect size+reach) of greater than or equal to 4 were presented to the RERP as the final product of this environmental scan. In total, 14 RCTs fit the above criteria for presentation to the RERP. Finally, a member of the team with experience in evidence-based evaluation (TJC) applied quality of evidence scoring to each RCT following the Grading of Recommendations, Assessment, Development, and Evaluation (GRADE) working group approach [28]. GRADE outcomes’ tables were created for the primary outcomes of each RCT (see tables in Multimedia Appendix 3).

### Step 2: Expert Panel Recruitment

#### 2a. Identify the Target Population to Conduct the Rapid Expert Review

The target population for the evidence review process can be local or national level, depending on goals for future implementation and effectiveness studies. Ideally, an initial national-level process to prioritize the most promising tools can be followed by local validation, which focuses much more on how high-priority tools need to be adapted to fit local clinical contexts and workflows. Targeting a national group of experts for initial prioritization has several advantages. First, a national panel lends itself to focusing on what might generally work to improve the patient experience rather than details of what might be practical in a particular context. Second, it allows the project team to obtain the perspective of clinical experts from multiple different geographic areas and a variety of clinical settings. Third, it allows health systems to incorporate expertise from beyond the boundaries of their own system, which enhances potential for solutions that can be used and evaluated at multiple institutions. Finally, a national panel allows health information technology (IT) companies developing technological interventions to evaluate the types of software most likely to be accepted by their customers.

The panel meeting of national experts can be held, if resources allow, at a national society meeting for practitioners in the area of interest to expand convenience for panelists and increase the number of experts willing and able to participate. The RERP meeting, focusing on the patients’ experience of cancer care, was held at the 2017 annual meeting of the American Society for Clinical Oncology (ASCO).

#### 2b. Recruit the Appropriate Mix of Participants

To identify potential panel participants, we looked for practitioners with expertise in the area of interest from across the nation. We sought to recruit panelists who practice in the field in which the intervention will be implemented and have first-hand knowledge of the topic and clinical workflows. We also considered whether to include patient representatives on the panel to provide insight into patient needs, preferences, and knowledge that can further inform the impact and feasibility of the technologies being considered. However, given the goal of evidentiary review at this stage, we chose to focus on clinical experts for this initial evidence review and prioritization. To select expert panelists, we first contacted national leaders within the domains of using technology in oncology care and improving the patient experience of cancer care. We asked these national leaders to nominate clinical oncologists with research or clinical interest in the patient experience of cancer care and PROs, such as monitoring and improving symptoms related to adverse effects of chemotherapy, cancer-related fatigue, comorbid depression, and anxiety. We then reached out to nominees to invite their participation. The final national panel comprised a convenience sample of clinical oncology leaders who were able to attend the annual meeting of ASCO in 2017. We identified 16 experts in medical oncology from across the United States, including physicians in both community oncology practices and academic medical centers. Our final panel consisted of 8 medical oncologists with a range of expertise relevant to the patient experience of cancer care and technology’s role in facilitating patient experience. Participants were recruited through direct contact by the principal investigator and coinvestigators and subsequent snowball sampling. If resources allow, panelists can be compensated for their time.

#### 2c. Introduce Participants to the Topic and Prepare Them for the Work Ahead

A short introductory meeting is helpful to set the tone for the expert panel, present background information, and allow panelists to ask questions and learn what to expect. For the introductory meeting, we held an hour-long teleconference 1 week before the RERP meeting in which we introduced ourselves and the panelists, gave the rationale for the project, and explained the panel members’ responsibility and what would take place during the RERP meeting.

The purpose of the RERP is for expert practitioners to evaluate the potential feasibility and impact of putting complex technological interventions into clinical practice. We defined the goal of our panel as helping oncologists and health systems nationwide evaluate the feasibility and impact of utilizing trial-tested PRO and SM tools to improve the patient experience of cancer care. On the basis of our decision to focus on symptoms common across many cancers, we asked participants to consider feasibility and impact for the *average patient* in the *average care setting*. We aimed to have applicability to the largest portion of oncology patients rather than focusing on rare or specialized cases.

### Step 3: Conduct the Rapid Evidence Review

We used a modified Delphi panel process. Modified Delphi panels are widely used in health research as a method to elicit group judgment that includes multiple rounds of rating, panelist discussion of judgment, and group facilitation to mitigate bias [29]. The modified Delphi was chosen as the best method of evaluation because it is seen as credible, widely used, and can quickly elicit expert consensus. Using the modified Delphi strategy, we conducted the RERP meeting in 3 parts: an introduction, initial rating, and rerating. We allowed time for discussion and questions in each part.

#### 3a. Creation of Study Summary Diagrams

**Figure 4:**
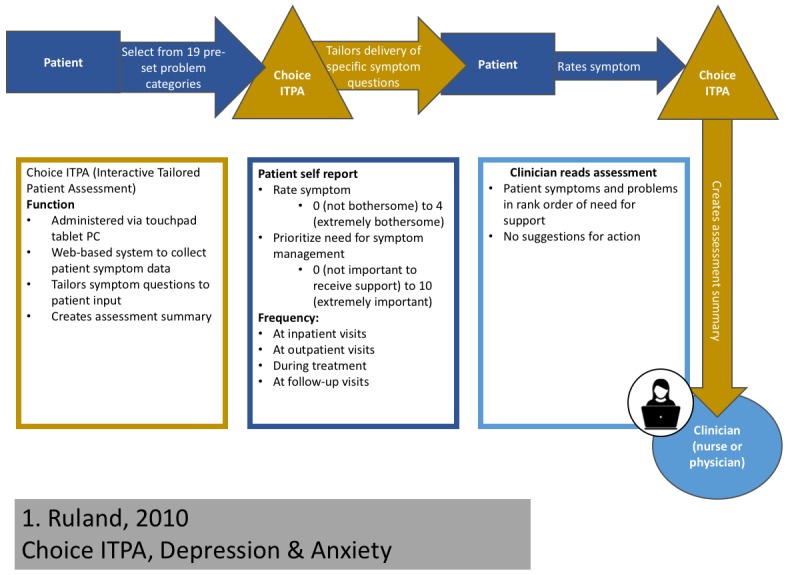
Example intervention diagram and flowchart. Diagram mapping out the Choice ITPA intervention in [30]. The patients selects from 18-preset problem categories via a Web-based system called Choice ITPA. The system tailors delivery of symptom questions based on patient responses. The patient rates their symptom, and the Choice ITPA system creates an assessment summary rank patient symptoms by priority, which is delivered to the clinician. Choice ITPA: Interactive Tailored Patient Assessment.

**Figure 5:**
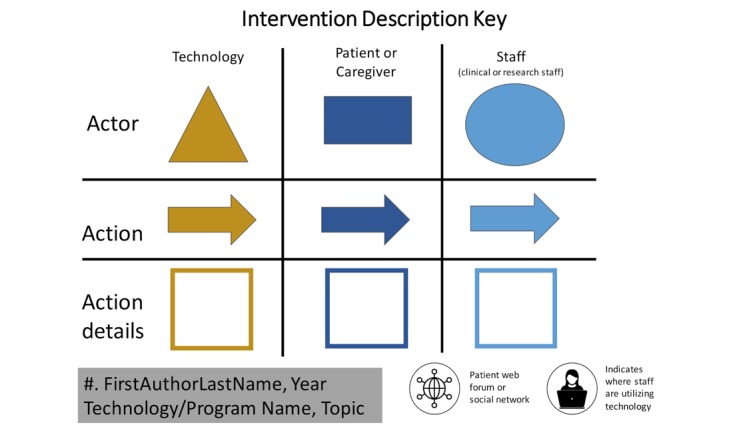
The key allows the intervention of any patient-reported outcome (PRO) or self-management (SM) tool to be mapped out, with a focus on the role of the technology, patient or caregiver, and staff (including providers).

For technological interventions in clinical care, there will likely be a standard set of *actors*, whereas the clinical actions may vary by intervention. Actors may include the technology, the patient or caregiver, or the clinician. When visualizing a complex intervention from a published trial, it is important to only include aspects that comprise the technological intervention and not aspects arising from the trial itself (eg, consent forms). Each actor will send, receive, and/or process information in some way. We presented the technology’s name and described its function in as much detail as possible. We described the frequency of patient contacts and detailed the information that patients provided to the technology or staff. In addition, we specified how the clinical team implemented the intervention. Symbols indicated whether staff interacted with technology and whether a social media network or patient forum was present (see Figure 4 for an example diagram [30] and Figure 5 for a key to the diagram).

**Impact criteria.**

Criteria 1: Impact

1. Evidence exists that using the intervention is likely to improve patient outcomes.
2. Actions are consistent with high-quality care.
3. Using the intervention is likely to affect many patients or have a significant impact on a smaller number of patients.
4. Intervention fills a gap: current rates of intervention’s actions are likely to be low.

**Feasibility criteria.**

Criteria 2: Feasibility

1. Actions are likely to be accepted by providers.
2. Actions fit with current workflows or workflows can be easily redesigned to fit.
3. Actions are consistent with current system incentives.
4. Actions will be accepted or welcomed by patients.

#### 3b. Rapid Evidence Review Panel Introduction

The goal of the RERP introduction is to remind the participants of the goals of the session and key concepts and terms and quickly set the stage for the focused discussion and rating that follows. Our project team led a brief introduction of the panel and its purpose. In addition, we defined feasibility and impact and how these concepts would be rated during the session (see Textboxes 1 and 2).

#### 3c. Rating

Maintaining a brisk pace is crucial to evaluate more than a handful of interventions. We allocated an average of 10 min per intervention; this included 4 min for material presentation, 4 min for clarifying questions, and 2 min for private rating. It is reasonable to expect that the first few interventions will take longer as panelists adjust to the specifics of the topic area and the panel structure. With the highly structured approach described below, our panel of medical oncologists was able to complete initial ratings of interventions from 14 RCTs in an average of 10 min per intervention.

What information is necessary to evaluate an intervention? Rapid evidence review requires highly structured information. For each intervention, the project team presented preprepared material, including a study synopsis, GRADE tables of evidence quality, and a visual description of the intervention. Examples of the structured materials are provided in the Web-based supplementary materials. To review each RCT, the panelists were shown a series of slides on a large projection screen. Slides included background information about each technological intervention and RCT, including how many patients used the technology in total and how many settings the system had been implemented in. Each panelist also had a binder with all information from the slides that they were able to reference throughout the review process. Table 1 shows a detailed description of all materials provided to each panelist (Table 1).

This structured information allows the panelists to quickly understand key aspects of the study and intervention, which they can then discuss while project staff takes notes on their comments. Finally, the panelists rated the intervention’s feasibility and its impact on a scale of 1 to 9, where 1 to 3 indicated low impact or feasibility, 4 to 6 indicated uncertain or equivocal impact or feasible, and 7 to 9 indicated highly feasible or high impact (Table 2) [28].

#### 3d. Rerating

The project team compiled the panelist’s ratings according to modified Delphi panel methods [31]. For each intervention, the project team presented the median score and counts for both feasibility and impact and indicated the level of panelist agreement (agree, disagree, or equivocal). After viewing their own and the group’s overall ratings and level-of-agreement, the panelists rediscussed the interventions. We prompted them to explain the rationale behind their initial rating, especially if it was higher or lower than the median. Research staff took notes on the discussion. Finally, the panelists completed a final rating of each intervention.

We followed the criteria outlined by Fitch et al to calculate agreement and disagreement [29]. For 8 panelists, counts indicated agreement when no more than 2 panelists rate the indication outside the 3-point region (1-3; 4-6; and 7-9) containing the median. Counts indicated disagreement when at least three panelists rate the indication in the 1 to 3 region *and* at least three panelists rate it in the 7 to 9 region. Otherwise, agreement level was determined to be equivocal. To accelerate the processing of ratings and levels of agreement for real-time use during the session, we prepared an Excel spreadsheet to automatically calculate and present median scores and counts to panelists.

**Table 1:** Materials for rapid evidence review panel (RERP) process.

Material	Purpose	Description	Project example	Source	Preparation time (per intervention)
Synopsis	Introduce the intervention and its context in a research study or guideline	A 5-sentence synopsis of the intervention and how it was originally tested or presented	Synopses were presented for all 14 RCTs^a^ based on the content of study abstracts	Study abstracts or society guidelines	5 min to standardize abstract content
GRADE^b^ tables	Evaluate quality of evidence and strength of recommendations	A common, sensible, and transparent approach to grading quality (or certainty) of evidence and strength of recommendations, which is now considered the standard in guideline development [28]	GRADE tables were used to evaluate quality of evidence for each of the 14 RCTs whose interventions panelists considered	GRADE working group [28]	10 min
Intervention flowchart	Present the intervention in a manner that allows for understanding of workflow impact, separate from study design	A standardized system for creating a visual representation of each intervention to describe the role of the technology, patient or caregiver, and clinician or research staff in the intervention	See Figures 4 and 5	Generated by research team from published manuscript	25 min
RE-AIM questions^c^	Encourage participants to evaluate aspects of interventions that would affect implementation	Four questions adapted from RE-AIM that address the ability to move research into action	Panelists were prompted to consider the following 4 questions after the presentation of each intervention: What are barriers to reaching the target population?; What are some unintended consequences of this intervention?; What are some barriers to adoption by sites and organizations?; and What are the staff and skills needed for implementation?	National Cancer Institute’s RTIPs RE-AIM scoring criteria [27]	<1 min
Published manuscript	Ability to reference original manuscripts for clarification	Full manuscript or guideline	Full published manuscripts of each RCT were made available for panelists during the RERP^e^ and were utilized several times to verify details of study design	Original manuscript or guideline	<1 min
Scales information	Ability to verify scale content, validity, and reliability	Scale items and reliability and validity information for all scales (those used in all interventions and study analyses)	Although we had scale information available, it was not used by panelists	Scales used were identified from the original manuscript and items, and reliability and validity were located from scale authors	30 min

^a^RCT: randomized controlled trial.

^b^GRADE: Grading of Recommendations, Assessment, Development, and Evaluation.

^c^RE-AIM: Reach, Effectiveness, Adoption, Implementation, and Maintenance Framework.

^d^RTIPs: Research-Tested Intervention Programs

^e^RERP: rapid evidence review panel.

#### 3e. Qualitative Supporting Information

Beyond prioritizing interventions quantitatively, understanding the rationale for panelists’ ratings can provide insights for local implementation. To collect these qualitative data, 2 members of the project team took notes during the discussion of key points by the panelists. Although recordings and transcripts are generally regarded as preferable for qualitative research [32], notes are preferable here because the time, effort, and resources required for transcription interfere with the goals of rapid analysis. Finally, we asked panelists for feedback on their participation in the RERP process using a short survey instrument, which included space for free text comments.

### Step 4: Analysis—How Are Expert Evaluations From the Rapid Evidence Review Panel Interpreted?

There are 3 tasks for data analysis: (4a) *prioritize interventions* for implementation, (4b) *identify features* of the interventions that contribute to positive or negative perceptions of feasibility or impact, and (4c) *identify perceived barriers to and facilitators for* putting the intervention into practice.

#### 4a. Prioritize

We ranked the interventions based on the panelists’ ratings. We determined this ranking by ordering the interventions according to the panel’s second (final) round of ratings using median scores and level of agreement for impact and feasibility. In determining the ranking, we weighted impact and feasibility equally and gave agreement second priority.

Depending on the project, stakeholders may consider the quantitative ranking described above as sufficient for determining which tools to prioritize for implementation. To decide whether this is sufficient, teams should consider the specificity required for meeting their aims. If simply recommending a *type* of system, then perhaps completing a prioritization is enough. However, if attempting to develop or implement a specific software and the team is interested in more specific details about *why* the rankings fall as they do, project teams should consider the benefits of additional analysis.

#### 4b. Identify Intervention Features

We also wanted to identify features of the interventions that contributed to positive or negative perceptions of the intervention’s feasibility and impact. This analysis allowed us to understand the types of tools and features that might be perceived as higher priority for implementation. We categorized each intervention as being primarily 1 of the 3 types: *SM Support*, *PROs*, and *communication.* Moreover, 2 interventions were classified with a secondary type. The project team generated a set of 17 codes to describe the features of the interventions; codes were generated from the original manuscript, and the diagrams designed to explain each intervention to the panelists and the panelists’ discussion. We then coded the RCTs based on which of the 17 features they possessed. All features were coded based on the information provided in the original manuscripts. We understand that scientific manuscripts do not contain full details of the computerized tools they describe and acknowledge that certain features or details are omitted in the RERP process. We also calculated the number of features described for each intervention. In addition, the notes from the panelists’ discussion were coded for presence of endorsement or opposition to each of the identified features, and the number of features endorsed or opposed was recorded for each intervention.

#### 4c. Identify Barriers and Facilitators

To identify perceived barriers to and facilitators for putting the interventions into practice, we conducted a content analysis of notes from the panel session using the Tailored Implementation for Chronic Disease (TICD) checklist [22]. The TICD checklist was developed from a systematic review of the literature in implementation science. It was designed to identify barriers and facilitators to implementation of health improvement interventions. Moreover, 2 members of the research staff read through discussion notes and coded per the TICD checklist. The raters then met and reconciled coding disagreements. From the final codes, themes were identified.

### Step 5: Local Validation Panel for Evaluation of Effectiveness

The purpose of the RERP process is to identify high-priority PRO and SM tools for further study and/or implementation. Although systematically identifying and prioritizing the most promising tools is an important first step, successfully implementing these complex tools will still require adaptations based on detailed knowledge of local workflows and context. After identifying SM and symptom tracker tools as effective, impactful, and feasible through the RERP process, we hosted a validation panel with a diverse set of stakeholders within our health system. These included hospital and clinical leadership, hematologists, oncologists, nurses, nurse educators, physician assistants, patient navigators, and other professionals from the University of Michigan Rogel Cancer Center. Using evidence summaries provided to them, these panelists were asked to validate, for locally focused purposes, the knowledge generated previously by a national panel of experts about the feasibility and impact of software tools intended to help improve the patient experience of cancer care. In addition, after having reviewed and commented on the scientific evidence about these tools and its meaning to a national group of oncologist experts, these panelists were asked to review and comment on an early design concept for a user customizable decision support app with features of SM and symptom tracker tools.

## Results

### Environmental Scan and Evaluation of Evidence Quality

We were able to rapidly review 14 manuscripts about computerized tools related to the OCM using our method. Panelists’ feedback indicated that participation was valuable and intuitive. What follows are the results we gained by doing so.

Our environmental scan and evidence review process yielded 14 RCT-tested interventions associated with at least moderate impact and reach. Evidence quality was variable, with most trial outcomes graded as being based on low to moderate quality evidence. Multimedia Appendix 3 provides the evidence review of the 14 RCTs from our environmental scan.

### Rapid Evidence Review Panel Step 4a: Prioritize

Participants rated interventions on impact and feasibility (Table 2). Agreement increased from the first to the second rating. Overall, most interventions were ranked more highly after discussion.

**Table 2:** Scoring schema for potential impact or feasibility and confidence in a given impact or feasibility rating.

Scores	Potential impact or feasibility	Confidence
7-9 (high)	High potential	Moderate to high (minor concerns only)
4-6 (equivocal)	Potential	Lower (major concerns)
1-3 (low)	No or low potential	—

**Table 3:** Outcomes of study ratings.

Study		Type	Agreement		Median		
			Rating 1	Rating 2	Rating 1	Rating 2	Final rank
**Feasibility**							
	1	PRO^a^	Equivocal	Equivocal	5	6	—
	2	PRO	Equivocal	Equivocal	3	4	—
	3	SM^b^	Agree	Agree	8	8	—
	4	SM or Communication	Agree	Agree	7	7	—
	5	PRO	Agree	Agree	5	4	—
	6	PRO	Equivocal	Equivocal	4	4	—
	7	PRO	Agree	Agree	6	6	—
	8	PRO	Equivocal	Equivocal	4	4	—
	9	PRO	Equivocal	Agree	6	5	—
	10	SM	Equivocal	Equivocal	7	7	—
	11	Communication or SM	Equivocal	Equivocal	4	4	—
	12	PRO	Equivocal	Equivocal	6	5	—
	13	SM	Equivocal	Agree	8	8	—
	14	SM	Agree	Agree	8	8	—
**Impact**							
	1	PRO	Equivocal	Equivocal	5	5	6
	2	PRO	Equivocal	Equivocal	7	7	3
	3	SM	Agree	Agree	8	8	1
	4	SM or Communication	Equivocal	Equivocal	7	7	2
	5	PRO	Equivocal	Agree	5	5	4
	6	PRO	Equivocal	Equivocal	6	6	6
	7	PRO	Equivocal	Equivocal	7	7	3
	8	PRO	Equivocal	Agree	5	5	5
	9	PRO	Equivocal	Equivocal	4	4	5
	10	SM	Equivocal	Agree	6	6	3
	11	Communication or SM	Equivocal	Equivocal	3	3	7
	12	PRO	Agree	Agree	6	5	5
	13	SM	Equivocal	Agree	7	7	1
	14	SM	Equivocal	Equivocal	7	7	2

^a^PRO: patient-reported outcome.

^b^SM: self-management.

**Table 4:** Average scores for feasibility and impact of different study designs based on second panelist’s ratings.

Study type	Feasibility	Impact
PRO^a^	4.72	5.23
SM^b^	7.18	6.64
Communication	5.07	5.00

^a^PRO: patient-reported outcome.

^b^SM: self-management.

### Rapid Evidence Review Panel Step 4b: Identify Intervention Features

We found that these complex interventions contained multiple features and that the panel had opinions about many of these features. We identified clear differences in how interventions were ranked based on the type of intervention being studied. We identified 3 main types of interventions among the 14 RCTs reviewed related to improving the patient experience of cancer care:

#### Self-Management (SM) Tools

Interventions with the primary function of providing resources and information to patients that involved no or limited clinician involvement supported patient SM and often provided patients with educational materials and were primarily patient facing.

#### Patient-Reported Outcomes (PROs)

Some of the interventions with the primary function of collecting PROs and transmitting that information to the clinician in some format to assist with treatment had additional components, such as decision support for patients and/or to help clinicians deal with the patient-reported information or functions to trigger notifications to clinicians when certain thresholds had been reached. These interventions contained both patient- and clinician-facing components.

#### Communication

Interventions with the primary function of facilitating patient-provider communication were both clinician- and patient-facing. Considering the ratings from 4a and the 3 main types of interventions identified in 4b (Table 3), SM support interventions consistently received highest rankings (average rating for feasibility=7.18 and average rating for impact=6.64; see Table 4).

### Rapid Evidence Review Panel Step 4c: Identify Implementation Barriers and Facilitators

Using the TICD framework, 6 major constructs were identified as barriers to implementation: quality of evidence, cultural appropriateness, patient behavior, availability of necessary resources, information systems, and payer or funder policies. No themes were identified as major facilitators. SM support interventions were seen as having fewer barriers to implementation, including being more appropriate for the workflow, more in line with patient behavior, carrying less legal risk, and having better evidence for their success.

Taken together, the results of this mixed-methods analysis allowed us to not only understand which specific interventions were considered most high priority but also to learn that SM support interventions may be perceived as more impactful and feasible to implement.

## Discussion

### Overview

The slow progress from research to practice is well documented [33,34]. The approach we describe here, a rapid evidence review for PRO and SM tools, is intended to balance the goals of rigor and efficiency for an evidence-based method to prioritize promising communication and decision-support technologies. Clinical experts found the evidence review structure to be engaging and the content sufficient to make judgments, and they were able to quickly and effectively prioritize a heterogeneous set of PRO and SM tools.

We observed that the RERP panel strongly favored implementation of SM and communication tools over PRO tools and indicated that this was largely because of less need for clinician involvement and lower legal risk. In addition, panelists expressed much skepticism about the feasibility of implementing PRO tools, despite high evidence of their success in the RCTs. We observed increased levels of agreement in the second round of rating, which is an expected feature of the modified Delphi process, after panelists come together and discuss the rationales for their initial ratings [31].

Health systems cannot put all effective tools into practice, no matter how promising. This prioritization process can be used by health systems and practices seeking to employ PRO and SM tools as the basis for local implementation studies or larger pragmatic effectiveness studies. Furthermore, the results of our evaluation highlight how our medical oncology experts favored SM support tools over tools utilizing PROs. This is surprising given what seems to be growing evidence of the effectiveness of these interventions to improve quality of life [35] and perhaps even lifespan [36]. Technological, workflow, cultural, and legal barriers caused our panel to evaluate these technologies as less feasible and impactful. Further evaluation of PRO and SM tools will help elucidate the extent to which these views about the challenges of implementing PRO-based tools are shared across institutions. Local evaluation can help clarify expectations and planning for implementation at individual institutions. Finally, the use of a systematic evidence review method such as that described here can help ensure that decision making for the implementation of new tools considers both the experience of relevant clinical experts and empirical findings from a diverse body of research literature. In addition, barriers exist that are because of the nature of PRO and SM tools and the research reporting process. Inadequate reporting of technology interventions makes evidence review difficult [37]. Furthermore, rapid technological change can outpace conducting and publishing RCTs, which further outpaces evidence review [38]. However, this further emphasizes the need for a rapid process to facilitate evidence being translated into practice as soon as the evidence is available.

### Limitations

Our rapid evaluation process has limitations. Although the RERP process was designed to limit the amount of time and resources it takes to complete the review and prioritization process, the time it takes to prepare the materials and synthesize evidence for the panelists is still nontrivial creating a potential barrier if resources are limited. However, a relatively small project team could follow our process and accomplish the majority of the work before convening the RERP. As the amount of evidence to review increases, the amount of preparation time needed may increase.

The focus of this particular rapid evidence review was on the clinician-facing aspects of existing tools, particularly the feasibility and impacts of integrating these complex technologies into clinical workflows. However, we recognize that this is only a first step in implementing new technologies. Fully successful implementation also requires incorporation of patient viewpoints [39-41]. For example, patient engagement is necessary to ensure that these tools strike the right balance between providing patients’ information and protecting their privacy [42]. Technology developers and health system leaders tasked with implementing and evaluating new tools need a robust process that incorporates all key stakeholders, including patients [39-41].

Although the RERP process provides a method to address the critical question, “Which PRO and SM tools should we prioritize for further study and implementation?,” it does not solve all of the challenges health systems face when seeking to use these complex tools to improve clinical care. It is likely that implementation challenges, both resulting from infrastructure limitations and clinician concerns, have limited utilization of these tools. To scale up use of PRO and SM tools in different clinical contexts nationally, a computational infrastructure that can support interoperable applications is necessary to support data collection and curation. PRO and SM tools may be an excellent use case for machine-encoded, computable biomedical knowledge curation, and execution platforms.

In the context of a relatively narrow and recent area of study, technology-based communication and support tools to improve the patient experience of cancer care, we identified numerous RCT-tested tools. To achieve the important task of improving the patient experience of cancer care, we needed a systematic and trustworthy process for identifying and prioritizing the most promising tools for further study and implementation.

Although health systems focused their efforts solely on tools with randomized trial evidence showing they can improve patient-important outcomes, the number of potential tools will likely exceed the system’s capacity to put them into practice. Moreover, these technologies can be complex. Integrating novel tools into clinical workflows has proven challenging [43]. Thus, even more than with other types of interventions, randomized trial’s evidence of the tool’s ability to improve outcomes may not translate into effectiveness in real-world settings. The RERP process presents a method to streamline the process of guideline review and data collection while maintaining a rigorous evidence-based grounding. We took advantage of multiple existing frameworks to streamline our process while maintaining rigor: current evidence searches and environmental scan procedures [17,44], the National Cancer Institute’s Research-Tested Intervention Program’s review process, the modified Delphi panel process, the GRADE ratings and summary of findings tables, and the TICD coding framework. Using these existing frameworks for each part of the evidence search and review process allowed for a systematic process that was feasible to complete within approximately 4 months.

In addition, the identification of potential useful features or perceived implementation barriers by experts (4b and 4c) may help health system leadership understand how the high-priority tools need to be adapted before implementation. The evaluation of the benefits and drawbacks of specific features of tools may inform the design or configuration of new technologies before implementation. For example, a system architect may consider deleting or modifying some features seen as barriers and including other features viewed as helpful. Thus, important next steps include taking the findings of an RERP to a local group of decision makers for validation and to determine how tools need to be adapted to fit a local context.

### Conclusions

Before PRO and SM tools, or other digital tools, may be broadly used, proper assessment of their potential feasibility and impact using an RERP process may be beneficial. The RERP process presented here may enable health care administrators to make more efficient and effective decisions about the implementation of novel technologies in clinical practice.

## Appendix

Multimedia Appendix 1 Symptom domains identified for the environmental scan.

Multimedia Appendix 2 Detailed information on environmental scan search strategy.

Multimedia Appendix 3 Grading of recommendations, assessment, development, and evaluation tables of evidence for each of the 14 randomized controlled trials selected for panelist review.

## Abbreviations

ASCO: American Society for Clinical Oncology
DoTTI: Design and develOpment, Testing early iterations, Testing for effectiveness, Integration, and implementation
GRADE: Grading of Recommendations, Assessment, Development, and Evaluation
IT: information technology
OCM: Oncology Care Model
PRO: patient-reported outcome
RCT: randomized controlled trial
RE-AIM: Reach, Effectiveness, Adoption, Implementation, and Maintenance Framework
RERP: Rapid Evidence Review Panel
RTIPs: Research-Tested Intervention Programs
SM: self-management
TICD: Tailored Implementation for Chronic Disease
