# Supplementary material for: A Rapid Process for Identifying and Prioritizing Technology-Based Tools for Health System Implementation
Source: JMIR Cancer. 2018 Nov 27;4(2):e11195. doi: 10.2196/11195 (PMC6290266; doi:10.2196/11195)
Supplement: Multimedia Appendix 1 [file cancer_v4i2e11195_app1.pdf]

Symptom Domains

- Pain assessment and management (OCM-4)
- Depression screening and follow-up plan (OCM-5)
- Anxiety screening and follow-up plan (OCM-6)
- Nutritional screening, appetite loss, intervention (OCM-6)
- Energy level, general health maintenance, QOL (OCM-6)
- Smoking cessation
- Self-care capability and social support
- Provider-to-patient communication
- Sexual health
- Chemotherapy-induced nausea and vomiting
