# Supplementary material for: A Rapid Process for Identifying and Prioritizing Technology-Based Tools for Health System Implementation
Source: JMIR Cancer. 2018 Nov 27;4(2):e11195. doi: 10.2196/11195 (PMC6290266; doi:10.2196/11195)
Supplement: Multimedia Appendix 2 [file cancer_v4i2e11195_app2.pdf]

Search Strategy

- Final database searches run in Ovid MEDLINE, Ovid MEDLINE In-Process & Other Non-Indexed Citations, Ovid MEDLINE ePub Ahead of Print. Preliminary searches run in the National Guideline Clearinghouse, PubMed, and MedlinePlus.gov to identify existing guidelines and patient-focused sources of relevant interventions
- Run on April 7, 2017
- Searches are focused
- Searches limited to English, 2008-2017 timeframe, randomized studies, adult studies. Two searches limited to 2013-2017 because of large result numbers
- Searches were conduced in two large groups, shown below. The first group identifies all studies with a technology intervention for the disease domains (anxiety, depression, etc.). The second group identifies all cancer studies with a technology intervention for the same domains. These two groups of searches are in separate tables. Each domain is a separate search within each table, and each table, then, consists of several final searches. In other words, there is a final search for anxiety & cancer & technology, a final search for depression & cancer & technology, etc.

The 'All' Search

#	Searches	Results
1	exp *information science/ or (technolog* or web or internet or email or e-mail or electronic mail or mail* or telephone* or smartphone* or mobile* or computer* or social network* or media or twitter or facebook or "2.0" or tele* or e-health* or ehealth* or digital* or digitiz* or texting or text messag* or self-monitor* or self-manag* or patient reported outcome* or online or cellphone* or phone* or audit* or feedback or assessment* or outreach or paper or scale* or questionnaire*).ti.	1836968
2	exp *depression/ or depression.ti. or depressive.ti. or depressed.ti.	136938
3	((and/1-2) and english.la. and randomi*.ti.) not ((adolescent/ or exp child/ or infant/) not exp adult/) not (pediatric* or paediatric*).ti.	482
4	limit 3 to yr="2013 -Current"	303
5	exp *anxiety/ or anxiety.ti.	55474
6	((and/1,5) and english.la. and randomi*.ti.) not ((adolescent/ or exp child/ or infant/) not exp adult/) not (pediatric* or paediatric*).ti.	214
7	limit 6 to yr="2008 -Current"	190
8	exp *"tobacco use cessation"/ or smoking cessation*.ti.	19807
9	((and/1,8) and english.la. and randomi*.ti.) not ((adolescent/ or exp child/ or infant/) not exp adult/) not (pediatric* or paediatric*).ti.	278
10	limit 9 to yr="2008 -Current"	238
1	exp *pain/ or pain.ti.	270757

11		
12	((and/1,11) and english.la. and randomi*.ti.) not ((adolescent/ or exp child/ or infant/) not exp adult/) not (pediatric* or paediatric*).ti.	470
13	limit 12 to yr="2013 -Current"	257
14	((chemotherap* and (nause* or vomit*)) or cinv).tw.	11095
15	((and/1,14) and english.la. and randomi*.ti.) not ((adolescent/ or exp child/ or infant/) not exp adult/) not (pediatric* or paediatric*).ti.	11
16	limit 15 to yr="2008 -Current"	6
17	exp anorexia/ or anorex*.tw. or appetite loss.tw. or ((eat* or feed* or food*) adj2 disorder*).tw.	45211
18	((and/1,17) and english.la. and randomi*.ti.) not ((adolescent/ or exp child/ or infant/) not exp adult/) not (pediatric* or paediatric*).ti.	53
19	limit 18 to yr="2008 -Current"	46
20	(caregiver adj3 (strain* or burden*)).tw. or (exp caregivers/ and (exp stress, psychological/ or (strain* or burden*).ti.))	7075
21	((and/1,20) and english.la. and randomi*.ti.) not ((adolescent/ or exp child/ or infant/) not exp adult/) not (pediatric* or paediatric*).ti.	32
22	limit 21 to yr="2008 -Current"	30
23	exp constipation/ or constipat*.tw.	24431
24	((and/1,23) and english.la. and randomi*.ti.) not ((adolescent/ or exp child/ or infant/) not exp adult/) not (pediatric* or paediatric*).ti.	39
25	limit 24 to yr="2008 -Current"	30
26	exp diarrhea/ or diarrhea*.tw.	93192
27	((and/1,26) and english.la. and randomi*.ti.) not ((adolescent/ or exp child/ or infant/) not exp adult/) not (pediatric* or paediatric*).ti.	37

28	limit 27 to yr="2008 -Current"	27
29	exp dyspnea/ or (dyspnea* or (short* adj3 breath*)).tw.	47508
30	((and/1,29) and english.la. and randomi*.ti.) not ((adolescent/ or exp child/ or infant/) not exp adult/) not (pediatric* or paediatric*).ti.	51
31	limit 30 to yr="2008 -Current"	37
32	exp fatigue/ or (fatigue* or tired).ti.	37766
33	((and/1,32) and english.la. and randomi*.ti.) not ((adolescent/ or exp child/ or infant/) not exp adult/) not (pediatric* or paediatric*).ti.	80
34	limit 33 to yr="2008 -Current"	76
35	exp *infection/ or exp *pneumonia/ or exp *fever/ or (infection* or fever* or pneumonia*).ti.	932089
36	((and/1,35) and english.la. and randomi*.ti.) not ((adolescent/ or exp child/ or infant/) not exp adult/) not (pediatric* or paediatric*).ti.	195
37	limit 36 to yr="2008 -Current"	147
38	exp sexual dysfunctions, psychological/ or (sexual or erectile or impoten* or dyspareun* or libido*).ti.	78943
39	((and/1,38) and english.la. and randomi*.ti.) not ((adolescent/ or exp child/ or infant/) not exp adult/) not (pediatric* or paediatric*).ti.	69
40	limit 39 to yr="2008 -Current"	53
41	exp sleep wake disorders/ or (insomnia* or sleep*).ti.	109903
42	((and/1,41) and english.la. and randomi*.ti.) not ((adolescent/ or exp child/ or infant/) not exp adult/) not (pediatric* or paediatric*).ti.	108
43	limit 42 to yr="2008 -Current"	92
4	(nutrition* or diet*).ti.	245545

44		
45	((and/1,44) and english.la. and randomi*.ti.) not ((adolescent/ or exp child/ or infant/) not exp adult/) not (pediatric* or paediatric*).ti.	135
46	limit 45 to yr="2008 -Current"	108
47	exp cognitive disorders/ or (cognitive adj2 (impair* or dysfunc* or disorder*)).tw.	62068
48	((and/1,47) and english.la. and randomi*.ti.) not ((adolescent/ or exp child/ or infant/) not exp adult/) not (pediatric* or paediatric*).ti.	90
49	limit 48 to yr="2008 -Current"	84
50	exp symptom assessment/ or (symptom* adj3 (assess* or evaluat* or monitor* or measur*)).ti.	4472
51	((and/1,50) and english.la. and randomi*.ti.) not ((adolescent/ or exp child/ or infant/) not exp adult/) not (pediatric* or paediatric*).ti.	18

Cancer Search

#	Searches	Results
1	exp neoplasms/ or (cancer* or neoplas* or tumor* or tumour* or carcinoma* or malignan*).ti.	3225812
2	exp *information science/ or (technolog* or web or internet or email or e-mail or electronic mail or mail* or telephone* or smartphone* or mobile* or computer* or social network* or media or twitter or facebook or "2.0" or tele* or e-health* or ehealth* or digital* or digitiz* or texting or text messag* or self-monitor* or self-manag* or patient reported outcome* or online or cellphone* or phone* or audit* or feedback or assessment* or decision* or outreach or paper or scale* or questionnaire*).ti.	1875802
3	exp depression/ or depress*.ti.	178800
4	((and/1-3) and english.la. and randomi*.ti.) not ((adolescent/ or exp child/ or infant/) not exp adult/) not (pediatric* or paediatric*).ti.	51
5	limit 4 to yr="2008 -Current"	46
6	exp anxiety/ or anxiety.ti.	88347

7	((and/1-2,6) and english.la. and randomi*.ti.) not ((adolescent/ or exp child/ or infant/) not exp adult/) not (pediatric* or paediatric*).ti.	62
8	limit 7 to yr="2008 -Current"	41
9	exp "tobacco use cessation"/ or smoking cessation*.tw.	33796
10	((and/1-2,9) and english.la. and randomi*.ti.) not ((adolescent/ or exp child/ or infant/) not exp adult/) not (pediatric* or paediatric*).ti.	14
11	limit 10 to yr="2008 -Current"	8
12	exp pain/ or pain*.ti.	397854
13	((and/1-2,12) and english.la. and randomi*.ti.) not ((adolescent/ or exp child/ or infant/) not exp adult/) not (pediatric* or paediatric*).ti.	58
14	limit 13 to yr="2008 -Current"	48
15	exp anorexia/ or anorex*.tw. or appetite loss.tw. or ((eat* or feed* or food*) adj2 disorder*).tw.	45200
16	((and/1-2,15) and english.la. and randomi*.ti.) not ((adolescent/ or exp child/ or infant/) not exp adult/) not (pediatric* or paediatric*).ti.	2
17	limit 16 to yr="2008 -Current"	2
18	(caregiver adj3 (strain* or burden*)).tw. or (exp caregivers/ and (exp stress, psychological/ or (strain* or burden*).ti.))	7075
19	((and/1-2,18) and english.la. and randomi*.ti.) not ((adolescent/ or exp child/ or infant/) not exp adult/) not (pediatric* or paediatric*).ti.	3
20	limit 19 to yr="2008 -Current"	3
21	exp constipation/ or constipat*.tw.	24426
22	((and/1-2,21) and english.la. and randomi*.ti.) not ((adolescent/ or exp child/ or infant/) not exp adult/) not (pediatric* or paediatric*).ti.	11
23	limit 22 to yr="2008 -Current"	11
2	exp diarrhea/ or diarrhea*.tw.	93174

24		
25	((and/1-2,24) and english.la. and randomi*.ti.) not ((adolescent/ or exp child/ or infant/) not exp adult/) not (pediatric* or paediatric*).ti.	7
26	limit 25 to yr="2008 -Current"	5
27	exp dyspnea/ or (dyspnea* or (short* adj3 breath*)).tw.	47498
28	((and/1-2,27) and english.la. and randomi*.ti.) not ((adolescent/ or exp child/ or infant/) not exp adult/) not (pediatric* or paediatric*).ti.	7
29	limit 28 to yr="2008 -Current"	6
30	exp fatigue/ or (fatigue* or tired).ti.	37759
31	((and/1-2,30) and english.la. and randomi*.ti.) not ((adolescent/ or exp child/ or infant/) not exp adult/) not (pediatric* or paediatric*).ti.	34
32	limit 31 to yr="2008 -Current"	32
33	exp infection/ or exp pneumonia/ or exp fever/ or (infection* or fever* or pneumonia*).tw.	1820699
34	((and/1-2,33) and english.la. and randomi*.ti.) not ((adolescent/ or exp child/ or infant/) not exp adult/) not (pediatric* or paediatric*).ti.	18
35	limit 34 to yr="2008 -Current"	13
36	exp sexual dysfunctions, psychological/ or (sexual or erectile or impoten* or dyspareun* or libido*).ti.	78920
37	((and/1-2,36) and english.la. and randomi*.ti.) not ((adolescent/ or exp child/ or infant/) not exp adult/) not (pediatric* or paediatric*).ti.	19
38	limit 37 to yr="2008 -Current"	16
39	exp sleep wake disorders/ or (insomnia* or sleep*).tw.	168615
40	((and/1-2,39) and english.la. and randomi*.ti.) not ((adolescent/ or exp child/ or infant/) not exp adult/) not (pediatric* or paediatric*).ti.	27

41	limit 40 to yr="2008 -Current"	24
42	(nutrition* or diet*).tw.	642357
43	((and/1-2,42) and english.la. and randomi*.ti.) not ((adolescent/ or exp child/ or infant/) not exp adult/) not (pediatric* or paediatric*).ti.	26
44	limit 43 to yr="2008 -Current"	19
45	exp cognitive disorders/ or (cognitive adj2 (impair* or dysfunc* or disorder*)).tw.	62016
46	((and/1-2,45) and english.la. and randomi*.ti.) not ((adolescent/ or exp child/ or infant/) not exp adult/) not (pediatric* or paediatric*).ti.	3
47	limit 46 to yr="2008 -Current"	3
48	exp symptom assessment/ or (symptom* adj3 (assess* or evaluat* or monitor* or measur*)).tw.	49149
49	((and/1-2,48) and english.la. and randomi*.ti.) not ((adolescent/ or exp child/ or infant/) not exp adult/) not (pediatric* or paediatric*).ti.	39
