# Supplementary material for: A Rapid Process for Identifying and Prioritizing Technology-Based Tools for Health System Implementation
Source: JMIR Cancer. 2018 Nov 27;4(2):e11195. doi: 10.2196/11195 (PMC6290266; doi:10.2196/11195)
Supplement: Multimedia Appendix 3 [file cancer_v4i2e11195_app3.pptx]

## Slide 1
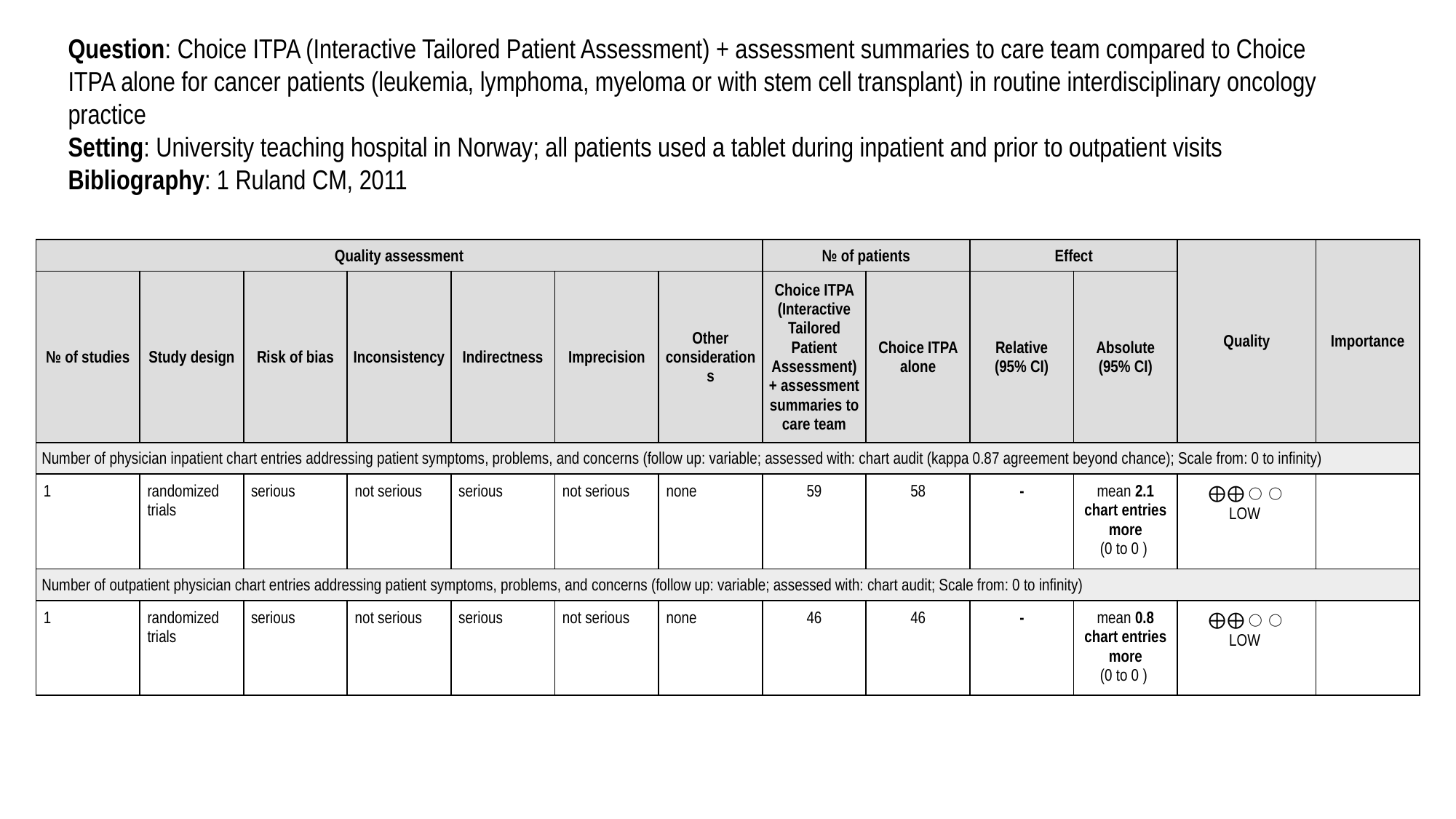

Question: Choice ITPA (Interactive Tailored Patient Assessment) + assessment summaries to care team compared to Choice ITPA alone for cancer patients (leukemia, lymphoma, myeloma or with stem cell transplant) in routine interdisciplinary oncology practice
Setting: University teaching hospital in Norway; all patients used a tablet during inpatient and prior to outpatient visits
Bibliography: 1 Ruland CM, 2011
| Quality assessment | | | | | | | № of patients | | Effect | | Quality | Importance |
| --- | --- | --- | --- | --- | --- | --- | --- | --- | --- | --- | --- | --- |
| № of studies | Study design | Risk of bias | Inconsistency | Indirectness | Imprecision | Other considerations | Choice ITPA (Interactive Tailored Patient Assessment) + assessment summaries to care team | Choice ITPA alone | Relative (95% CI) | Absolute (95% CI) | | |
| Number of physician inpatient chart entries addressing patient symptoms, problems, and concerns (follow up: variable; assessed with: chart audit (kappa 0.87 agreement beyond chance); Scale from: 0 to infinity) | | | | | | | | | | | | |
| 1 | randomized trials | serious | not serious | serious | not serious | none | 59 | 58 | - | mean 2.1 chart entries more (0 to 0 ) | ⨁⨁◯◯ LOW | |
| Number of outpatient physician chart entries addressing patient symptoms, problems, and concerns (follow up: variable; assessed with: chart audit; Scale from: 0 to infinity) | | | | | | | | | | | | |
| 1 | randomized trials | serious | not serious | serious | not serious | none | 46 | 46 | - | mean 0.8 chart entries more (0 to 0 ) | ⨁⨁◯◯ LOW | |

## Slide 2
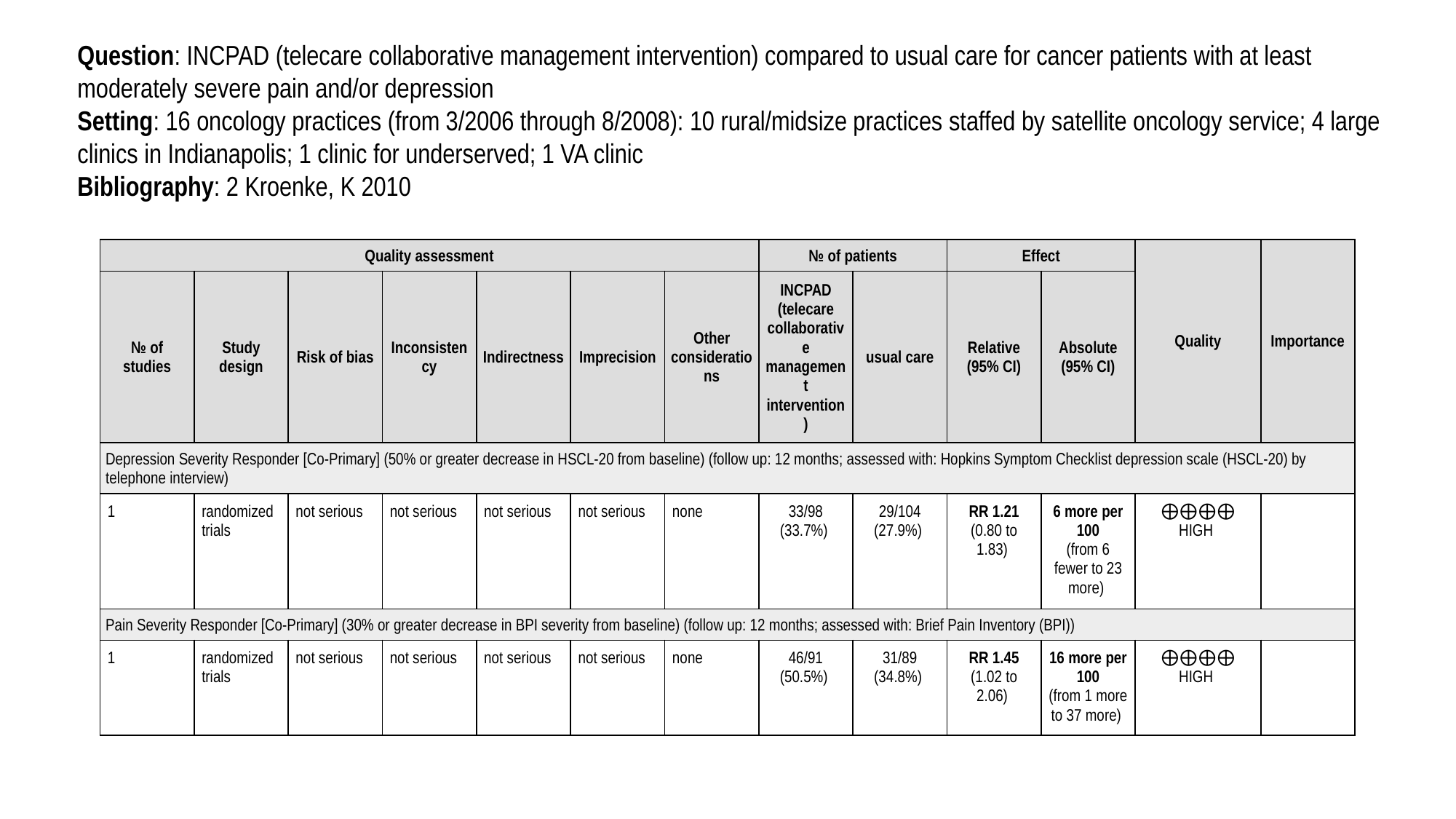

Question: INCPAD (telecare collaborative management intervention) compared to usual care for cancer patients with at least moderately severe pain and/or depression
Setting: 16 oncology practices (from 3/2006 through 8/2008): 10 rural/midsize practices staffed by satellite oncology service; 4 large clinics in Indianapolis; 1 clinic for underserved; 1 VA clinic
Bibliography: 2 Kroenke, K 2010
| Quality assessment | | | | | | | № of patients | | Effect | | Quality | Importance |
| --- | --- | --- | --- | --- | --- | --- | --- | --- | --- | --- | --- | --- |
| № of studies | Study design | Risk of bias | Inconsistency | Indirectness | Imprecision | Other considerations | INCPAD (telecare collaborative management intervention) | usual care | Relative (95% CI) | Absolute (95% CI) | | |
| Depression Severity Responder [Co-Primary] (50% or greater decrease in HSCL-20 from baseline) (follow up: 12 months; assessed with: Hopkins Symptom Checklist depression scale (HSCL-20) by telephone interview) | | | | | | | | | | | | |
| 1 | randomized trials | not serious | not serious | not serious | not serious | none | 33/98 (33.7%) | 29/104 (27.9%) | RR 1.21 (0.80 to 1.83) | 6 more per 100 (from 6 fewer to 23 more) | ⨁⨁⨁⨁ HIGH | |
| Pain Severity Responder [Co-Primary] (30% or greater decrease in BPI severity from baseline) (follow up: 12 months; assessed with: Brief Pain Inventory (BPI)) | | | | | | | | | | | | |
| 1 | randomized trials | not serious | not serious | not serious | not serious | none | 46/91 (50.5%) | 31/89 (34.8%) | RR 1.45 (1.02 to 2.06) | 16 more per 100 (from 1 more to 37 more) | ⨁⨁⨁⨁ HIGH | |

## Slide 3
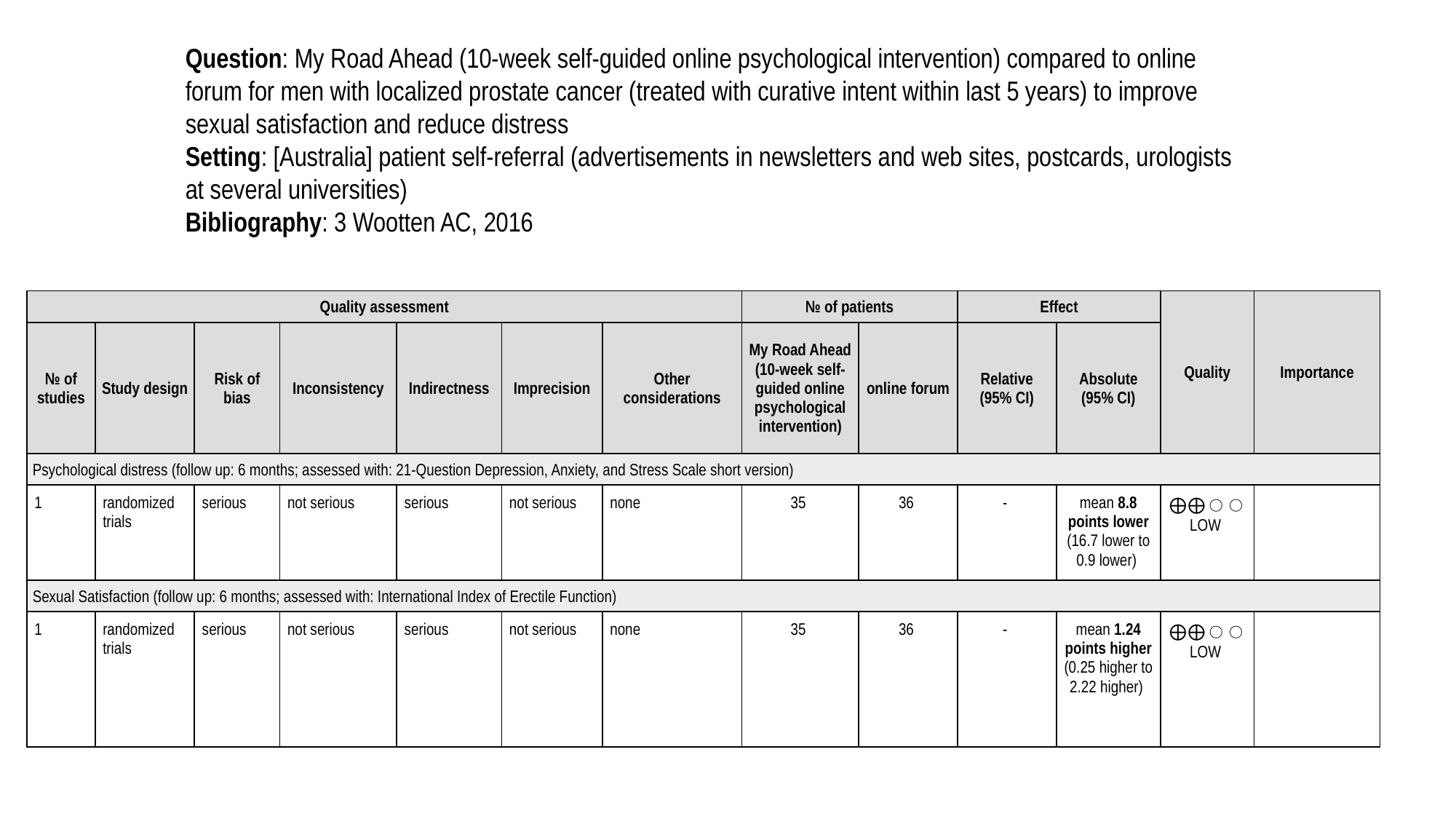

Question: My Road Ahead (10-week self-guided online psychological intervention) compared to online forum for men with localized prostate cancer (treated with curative intent within last 5 years) to improve sexual satisfaction and reduce distress
Setting: [Australia] patient self-referral (advertisements in newsletters and web sites, postcards, urologists at several universities)
Bibliography: 3 Wootten AC, 2016
| Quality assessment | | | | | | | № of patients | | Effect | | Quality | Importance |
| --- | --- | --- | --- | --- | --- | --- | --- | --- | --- | --- | --- | --- |
| № of studies | Study design | Risk of bias | Inconsistency | Indirectness | Imprecision | Other considerations | My Road Ahead (10-week self-guided online psychological intervention) | online forum | Relative (95% CI) | Absolute (95% CI) | | |
| Psychological distress (follow up: 6 months; assessed with: 21-Question Depression, Anxiety, and Stress Scale short version) | | | | | | | | | | | | |
| 1 | randomized trials | serious | not serious | serious | not serious | none | 35 | 36 | - | mean 8.8 points lower (16.7 lower to 0.9 lower) | ⨁⨁◯◯ LOW | |
| Sexual Satisfaction (follow up: 6 months; assessed with: International Index of Erectile Function) | | | | | | | | | | | | |
| 1 | randomized trials | serious | not serious | serious | not serious | none | 35 | 36 | - | mean 1.24 points higher (0.25 higher to 2.22 higher) | ⨁⨁◯◯ LOW | |

## Slide 4
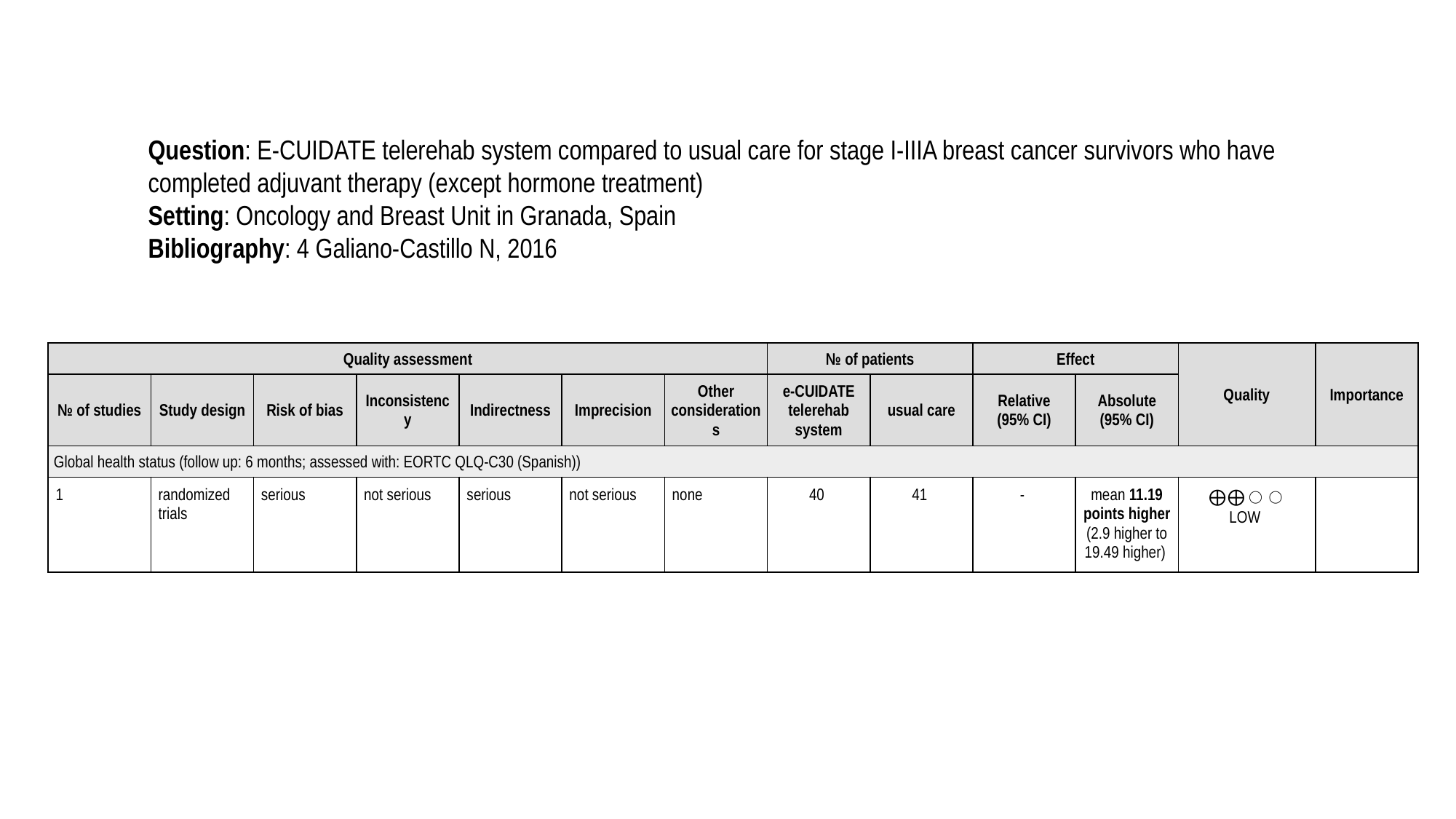

Question: E-CUIDATE telerehab system compared to usual care for stage I-IIIA breast cancer survivors who have completed adjuvant therapy (except hormone treatment)
Setting: Oncology and Breast Unit in Granada, Spain
Bibliography: 4 Galiano-Castillo N, 2016
| Quality assessment | | | | | | | № of patients | | Effect | | Quality | Importance |
| --- | --- | --- | --- | --- | --- | --- | --- | --- | --- | --- | --- | --- |
| № of studies | Study design | Risk of bias | Inconsistency | Indirectness | Imprecision | Other considerations | e-CUIDATE telerehab system | usual care | Relative (95% CI) | Absolute (95% CI) | | |
| Global health status (follow up: 6 months; assessed with: EORTC QLQ-C30 (Spanish)) | | | | | | | | | | | | |
| 1 | randomized trials | serious | not serious | serious | not serious | none | 40 | 41 | - | mean 11.19 points higher (2.9 higher to 19.49 higher) | ⨁⨁◯◯ LOW | |

## Slide 5
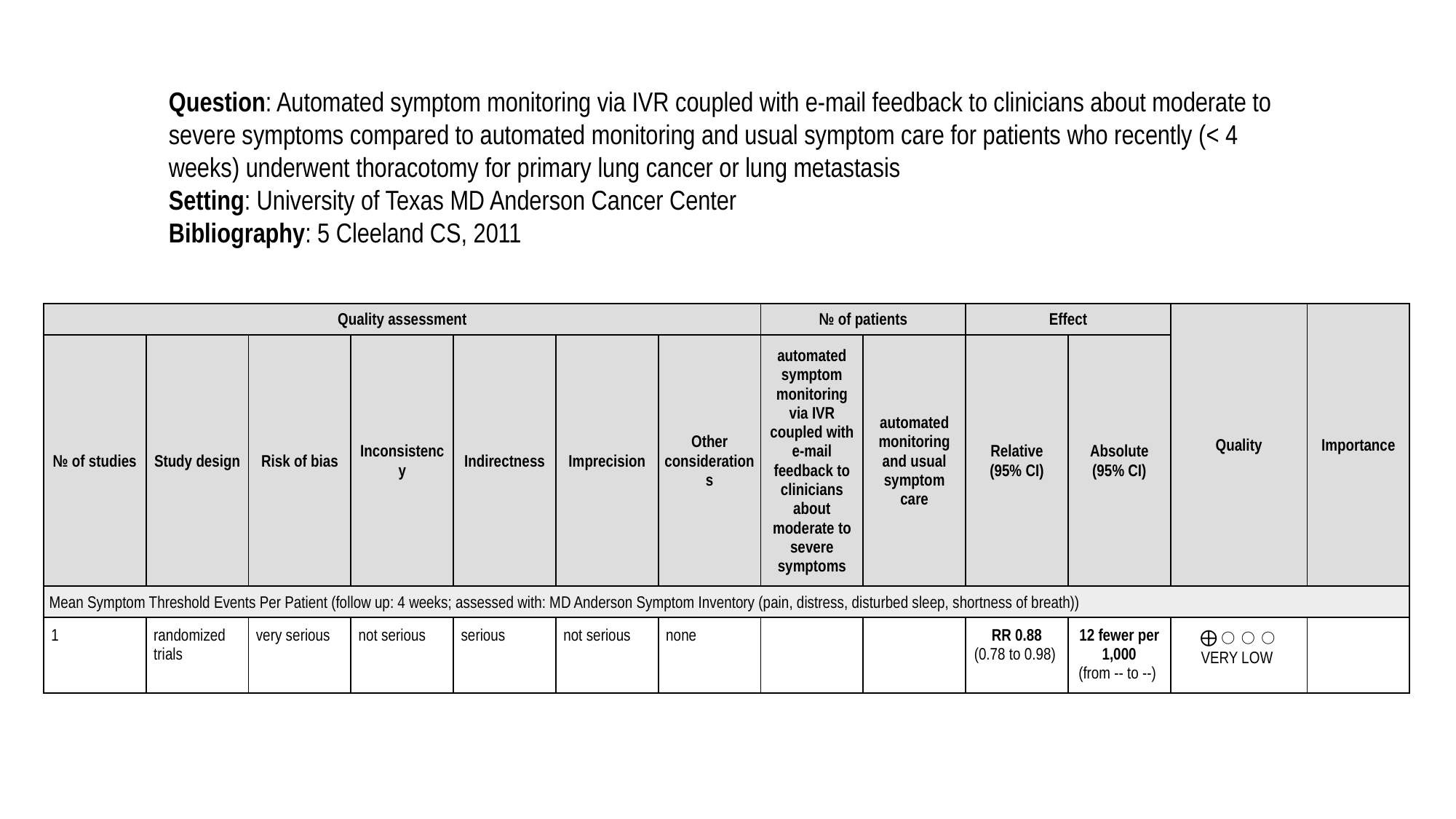

Question: Automated symptom monitoring via IVR coupled with e-mail feedback to clinicians about moderate to severe symptoms compared to automated monitoring and usual symptom care for patients who recently (< 4 weeks) underwent thoracotomy for primary lung cancer or lung metastasis
Setting: University of Texas MD Anderson Cancer Center
Bibliography: 5 Cleeland CS, 2011
| Quality assessment | | | | | | | № of patients | | Effect | | Quality | Importance |
| --- | --- | --- | --- | --- | --- | --- | --- | --- | --- | --- | --- | --- |
| № of studies | Study design | Risk of bias | Inconsistency | Indirectness | Imprecision | Other considerations | automated symptom monitoring via IVR coupled with e-mail feedback to clinicians about moderate to severe symptoms | automated monitoring and usual symptom care | Relative (95% CI) | Absolute (95% CI) | | |
| Mean Symptom Threshold Events Per Patient (follow up: 4 weeks; assessed with: MD Anderson Symptom Inventory (pain, distress, disturbed sleep, shortness of breath)) | | | | | | | | | | | | |
| 1 | randomized trials | very serious | not serious | serious | not serious | none | | | RR 0.88 (0.78 to 0.98) | 12 fewer per 1,000 (from -- to --) | ⨁◯◯◯ VERY LOW | |

## Slide 6
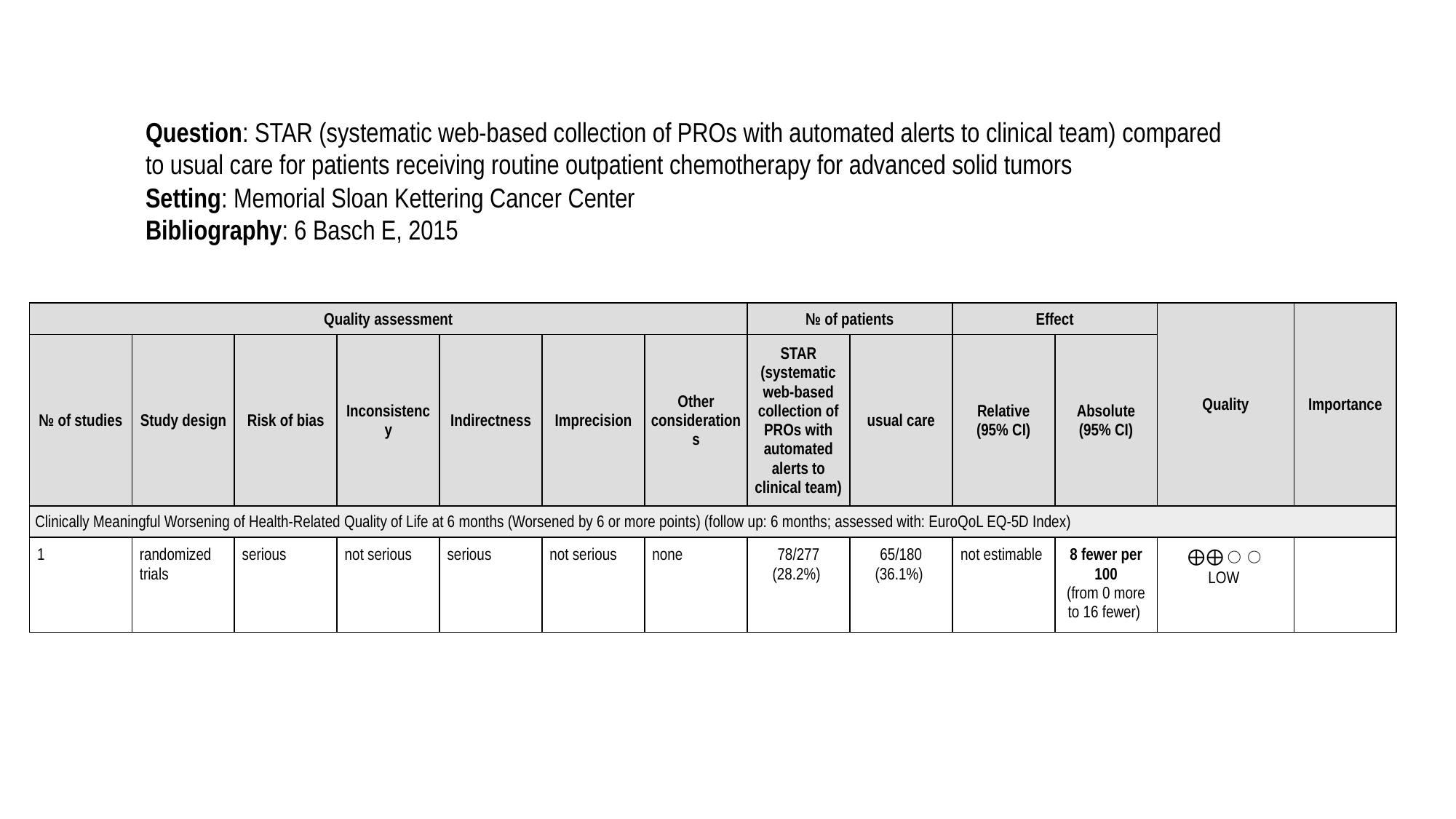

Question: STAR (systematic web-based collection of PROs with automated alerts to clinical team) compared to usual care for patients receiving routine outpatient chemotherapy for advanced solid tumors
Setting: Memorial Sloan Kettering Cancer Center
Bibliography: 6 Basch E, 2015
| Quality assessment | | | | | | | № of patients | | Effect | | Quality | Importance |
| --- | --- | --- | --- | --- | --- | --- | --- | --- | --- | --- | --- | --- |
| № of studies | Study design | Risk of bias | Inconsistency | Indirectness | Imprecision | Other considerations | STAR (systematic web-based collection of PROs with automated alerts to clinical team) | usual care | Relative (95% CI) | Absolute (95% CI) | | |
| Clinically Meaningful Worsening of Health-Related Quality of Life at 6 months (Worsened by 6 or more points) (follow up: 6 months; assessed with: EuroQoL EQ-5D Index) | | | | | | | | | | | | |
| 1 | randomized trials | serious | not serious | serious | not serious | none | 78/277 (28.2%) | 65/180 (36.1%) | not estimable | 8 fewer per 100 (from 0 more to 16 fewer) | ⨁⨁◯◯ LOW | |

## Slide 7
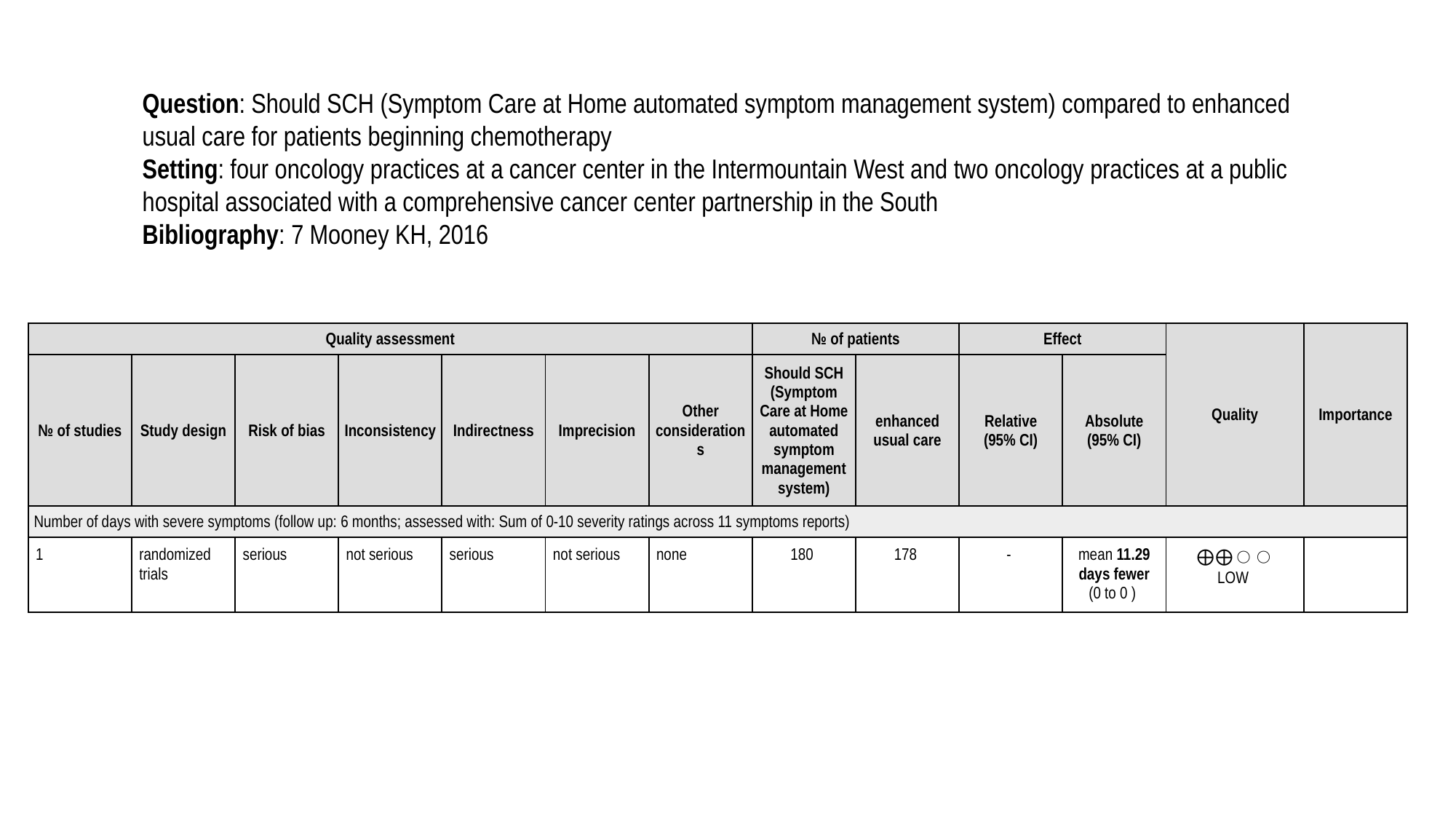

Question: Should SCH (Symptom Care at Home automated symptom management system) compared to enhanced usual care for patients beginning chemotherapy
Setting: four oncology practices at a cancer center in the Intermountain West and two oncology practices at a public hospital associated with a comprehensive cancer center partnership in the South
Bibliography: 7 Mooney KH, 2016
| Quality assessment | | | | | | | № of patients | | Effect | | Quality | Importance |
| --- | --- | --- | --- | --- | --- | --- | --- | --- | --- | --- | --- | --- |
| № of studies | Study design | Risk of bias | Inconsistency | Indirectness | Imprecision | Other considerations | Should SCH (Symptom Care at Home automated symptom management system) | enhanced usual care | Relative (95% CI) | Absolute (95% CI) | | |
| Number of days with severe symptoms (follow up: 6 months; assessed with: Sum of 0-10 severity ratings across 11 symptoms reports) | | | | | | | | | | | | |
| 1 | randomized trials | serious | not serious | serious | not serious | none | 180 | 178 | - | mean 11.29 days fewer (0 to 0 ) | ⨁⨁◯◯ LOW | |

## Slide 8
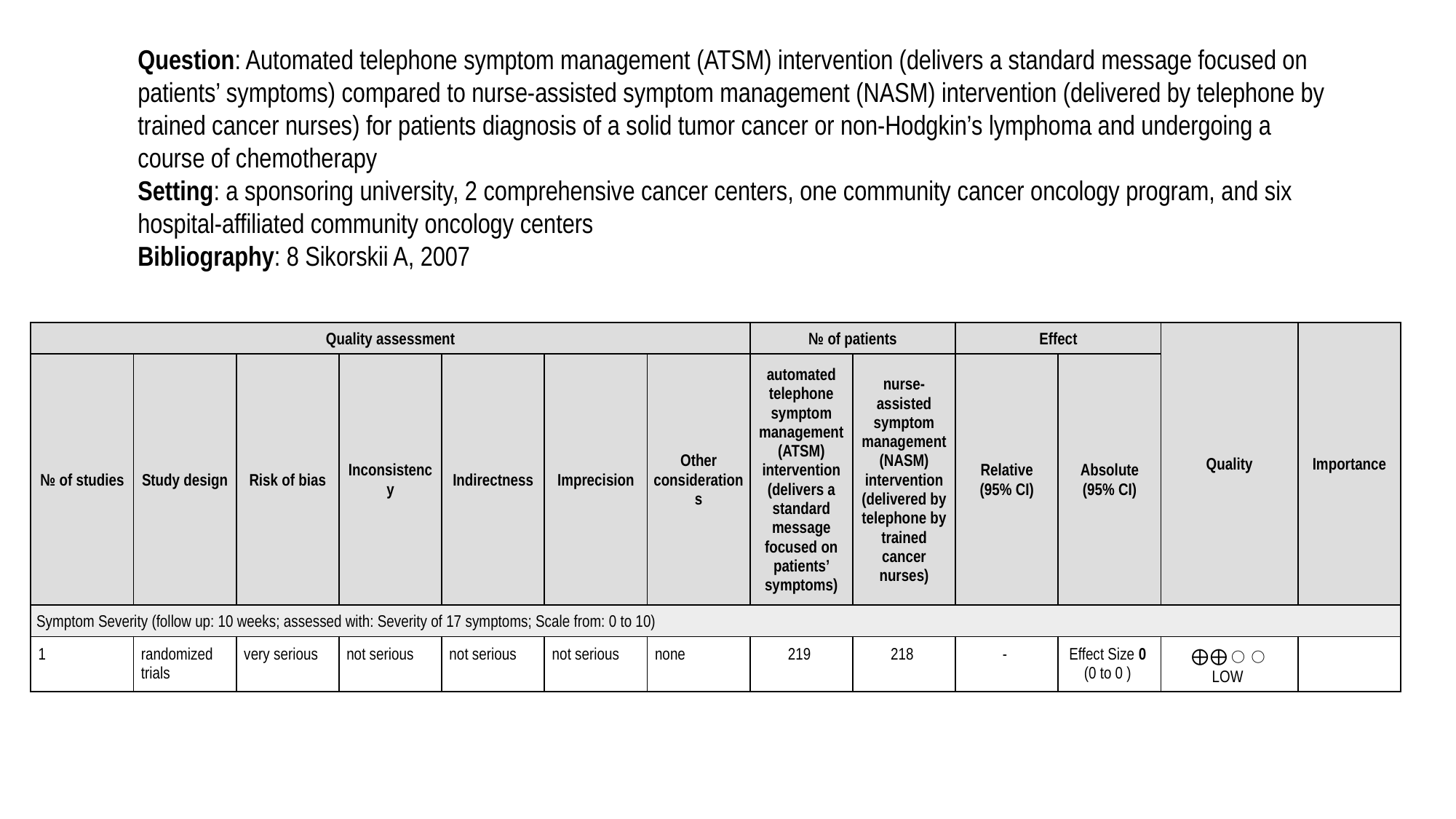

Question: Automated telephone symptom management (ATSM) intervention (delivers a standard message focused on patients’ symptoms) compared to nurse-assisted symptom management (NASM) intervention (delivered by telephone by trained cancer nurses) for patients diagnosis of a solid tumor cancer or non-Hodgkin’s lymphoma and undergoing a course of chemotherapy
Setting: a sponsoring university, 2 comprehensive cancer centers, one community cancer oncology program, and six hospital-affiliated community oncology centers
Bibliography: 8 Sikorskii A, 2007
| Quality assessment | | | | | | | № of patients | | Effect | | Quality | Importance |
| --- | --- | --- | --- | --- | --- | --- | --- | --- | --- | --- | --- | --- |
| № of studies | Study design | Risk of bias | Inconsistency | Indirectness | Imprecision | Other considerations | automated telephone symptom management (ATSM) intervention (delivers a standard message focused on patients’ symptoms) | nurse-assisted symptom management (NASM) intervention (delivered by telephone by trained cancer nurses) | Relative (95% CI) | Absolute (95% CI) | | |
| Symptom Severity (follow up: 10 weeks; assessed with: Severity of 17 symptoms; Scale from: 0 to 10) | | | | | | | | | | | | |
| 1 | randomized trials | very serious | not serious | not serious | not serious | none | 219 | 218 | - | Effect Size 0 (0 to 0 ) | ⨁⨁◯◯ LOW | |

## Slide 9
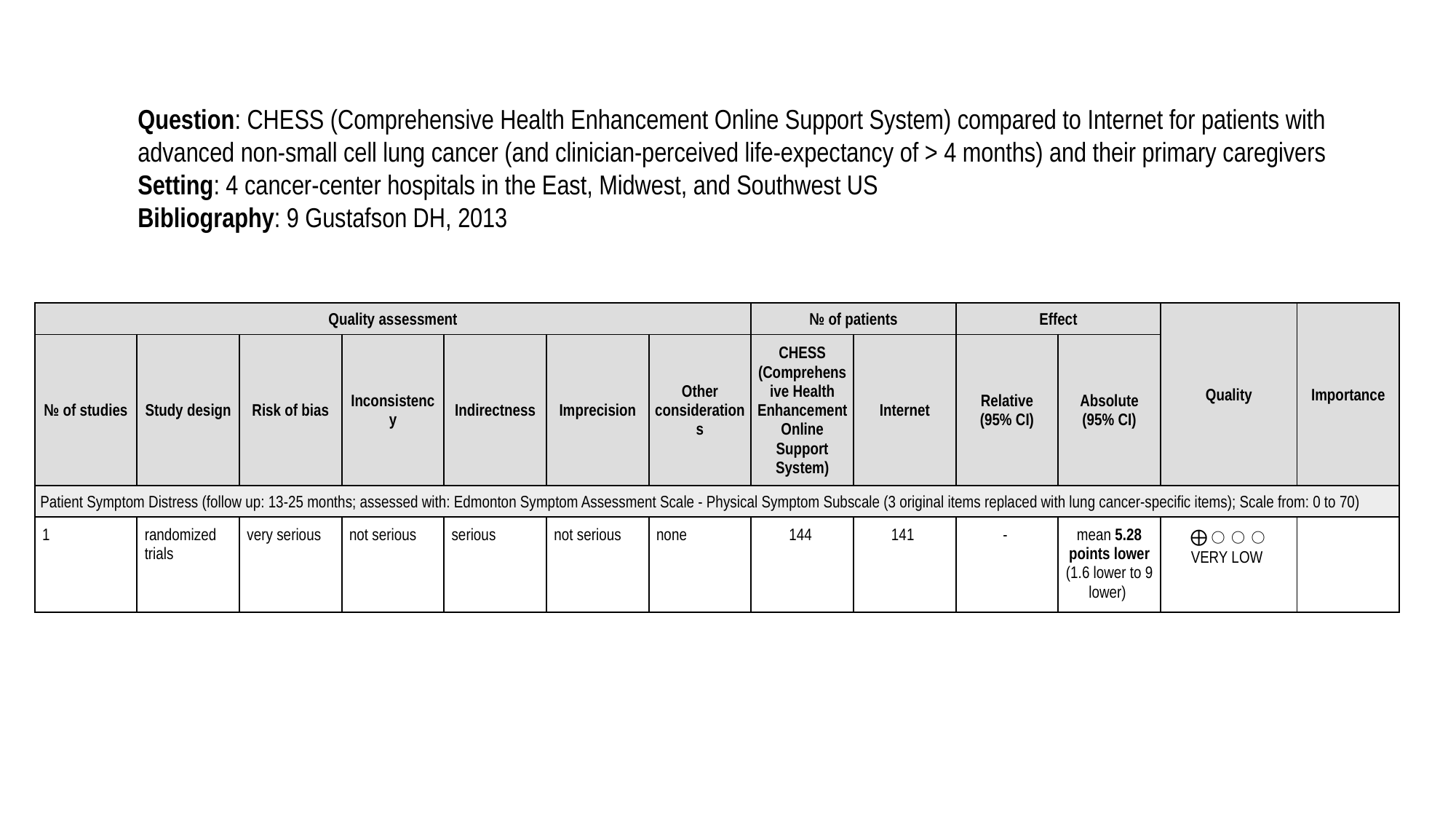

Question: CHESS (Comprehensive Health Enhancement Online Support System) compared to Internet for patients with advanced non-small cell lung cancer (and clinician-perceived life-expectancy of > 4 months) and their primary caregivers
Setting: 4 cancer-center hospitals in the East, Midwest, and Southwest US
Bibliography: 9 Gustafson DH, 2013
| Quality assessment | | | | | | | № of patients | | Effect | | Quality | Importance |
| --- | --- | --- | --- | --- | --- | --- | --- | --- | --- | --- | --- | --- |
| № of studies | Study design | Risk of bias | Inconsistency | Indirectness | Imprecision | Other considerations | CHESS (Comprehensive Health Enhancement Online Support System) | Internet | Relative (95% CI) | Absolute (95% CI) | | |
| Patient Symptom Distress (follow up: 13-25 months; assessed with: Edmonton Symptom Assessment Scale - Physical Symptom Subscale (3 original items replaced with lung cancer-specific items); Scale from: 0 to 70) | | | | | | | | | | | | |
| 1 | randomized trials | very serious | not serious | serious | not serious | none | 144 | 141 | - | mean 5.28 points lower (1.6 lower to 9 lower) | ⨁◯◯◯ VERY LOW | |

## Slide 10
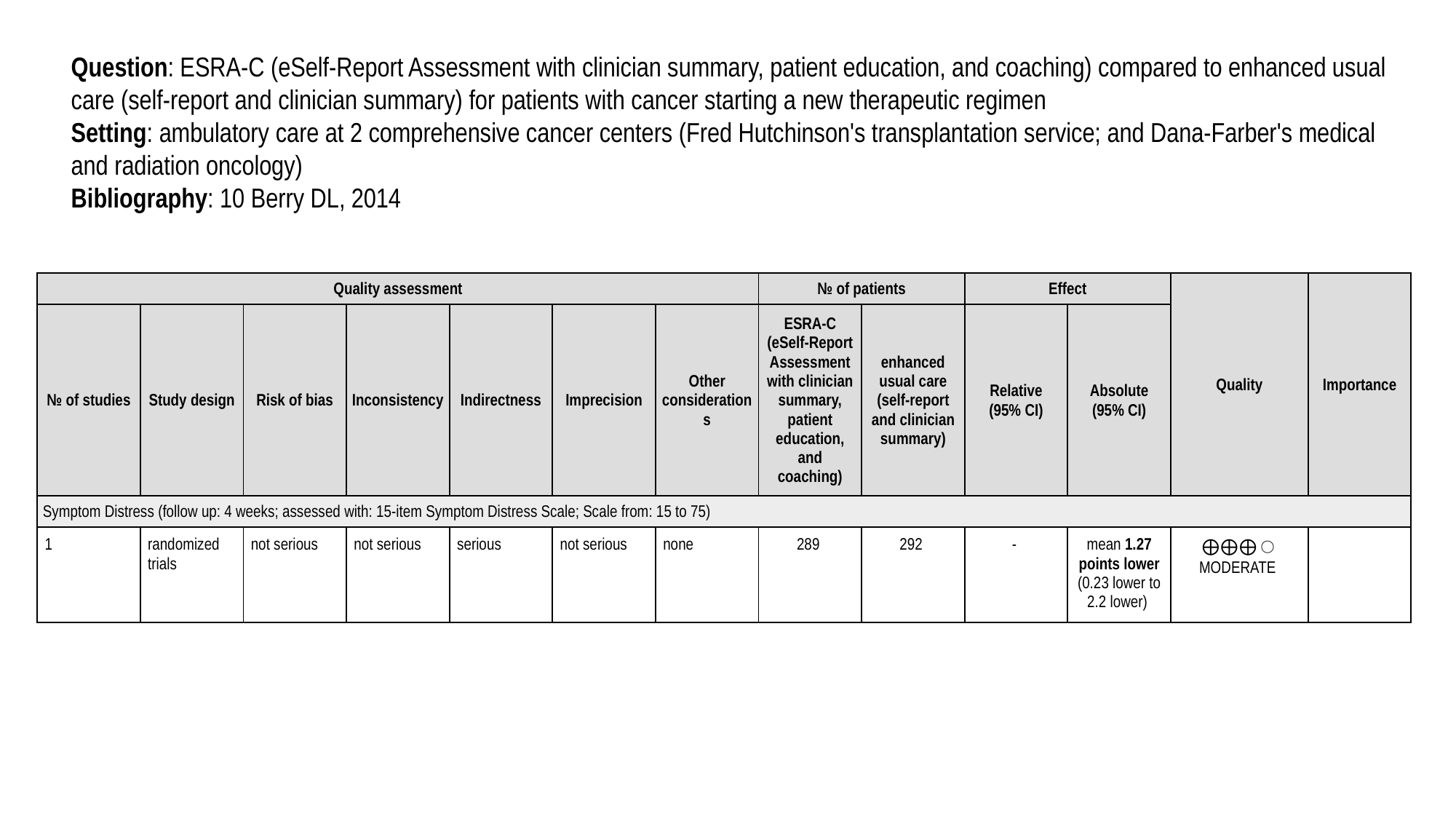

Question: ESRA-C (eSelf-Report Assessment with clinician summary, patient education, and coaching) compared to enhanced usual care (self-report and clinician summary) for patients with cancer starting a new therapeutic regimen
Setting: ambulatory care at 2 comprehensive cancer centers (Fred Hutchinson's transplantation service; and Dana-Farber's medical and radiation oncology)
Bibliography: 10 Berry DL, 2014
| Quality assessment | | | | | | | № of patients | | Effect | | Quality | Importance |
| --- | --- | --- | --- | --- | --- | --- | --- | --- | --- | --- | --- | --- |
| № of studies | Study design | Risk of bias | Inconsistency | Indirectness | Imprecision | Other considerations | ESRA-C (eSelf-Report Assessment with clinician summary, patient education, and coaching) | enhanced usual care (self-report and clinician summary) | Relative (95% CI) | Absolute (95% CI) | | |
| Symptom Distress (follow up: 4 weeks; assessed with: 15-item Symptom Distress Scale; Scale from: 15 to 75) | | | | | | | | | | | | |
| 1 | randomized trials | not serious | not serious | serious | not serious | none | 289 | 292 | - | mean 1.27 points lower (0.23 lower to 2.2 lower) | ⨁⨁⨁◯ MODERATE | |

## Slide 11
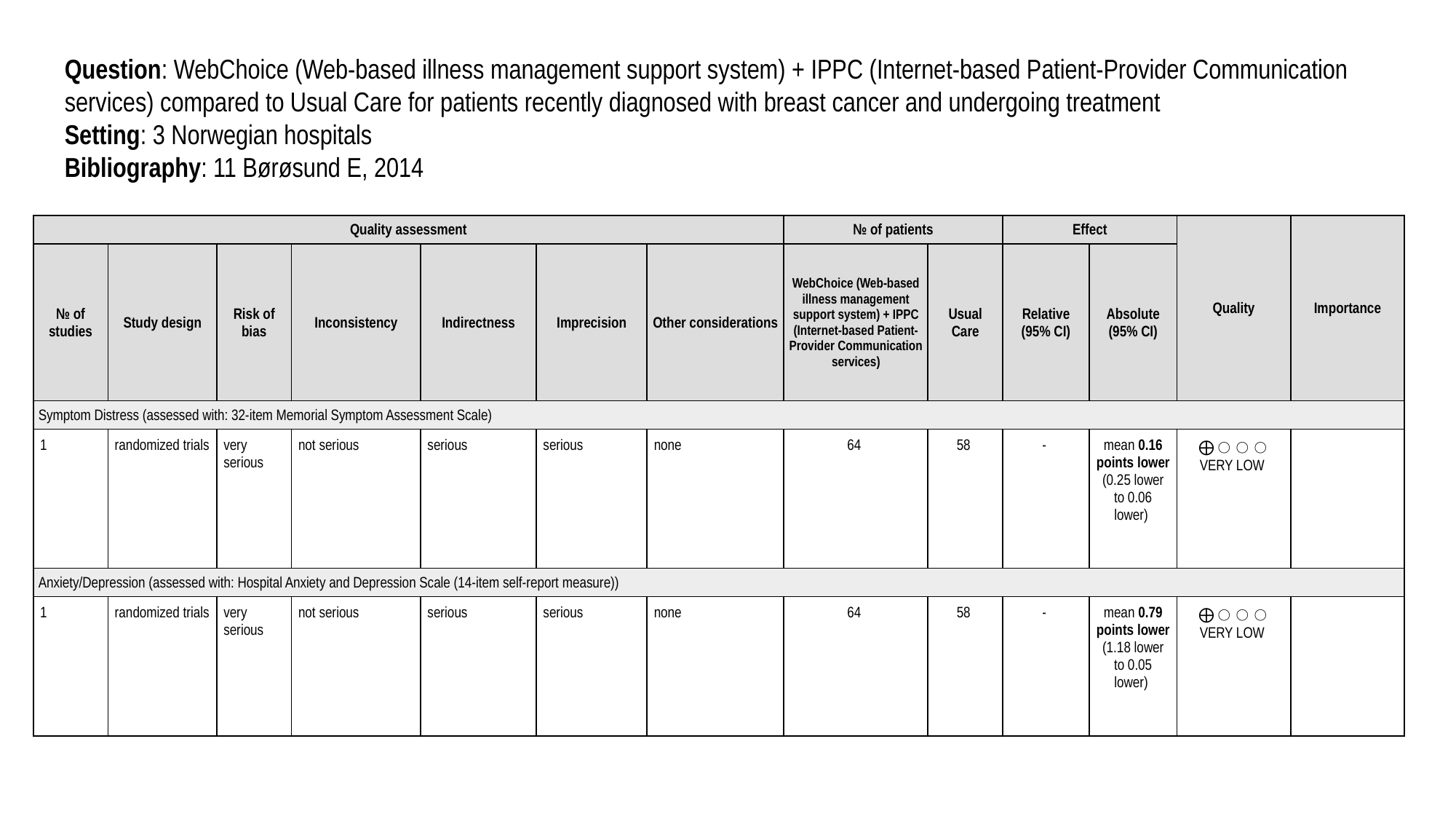

Question: WebChoice (Web-based illness management support system) + IPPC (Internet-based Patient-Provider Communication services) compared to Usual Care for patients recently diagnosed with breast cancer and undergoing treatment
Setting: 3 Norwegian hospitals
Bibliography: 11 Børøsund E, 2014
| Quality assessment | | | | | | | № of patients | | Effect | | Quality | Importance |
| --- | --- | --- | --- | --- | --- | --- | --- | --- | --- | --- | --- | --- |
| № of studies | Study design | Risk of bias | Inconsistency | Indirectness | Imprecision | Other considerations | WebChoice (Web-based illness management support system) + IPPC (Internet-based Patient-Provider Communication services) | Usual Care | Relative (95% CI) | Absolute (95% CI) | | |
| Symptom Distress (assessed with: 32-item Memorial Symptom Assessment Scale) | | | | | | | | | | | | |
| 1 | randomized trials | very serious | not serious | serious | serious | none | 64 | 58 | - | mean 0.16 points lower (0.25 lower to 0.06 lower) | ⨁◯◯◯ VERY LOW | |
| Anxiety/Depression (assessed with: Hospital Anxiety and Depression Scale (14-item self-report measure)) | | | | | | | | | | | | |
| 1 | randomized trials | very serious | not serious | serious | serious | none | 64 | 58 | - | mean 0.79 points lower (1.18 lower to 0.05 lower) | ⨁◯◯◯ VERY LOW | |

## Slide 12
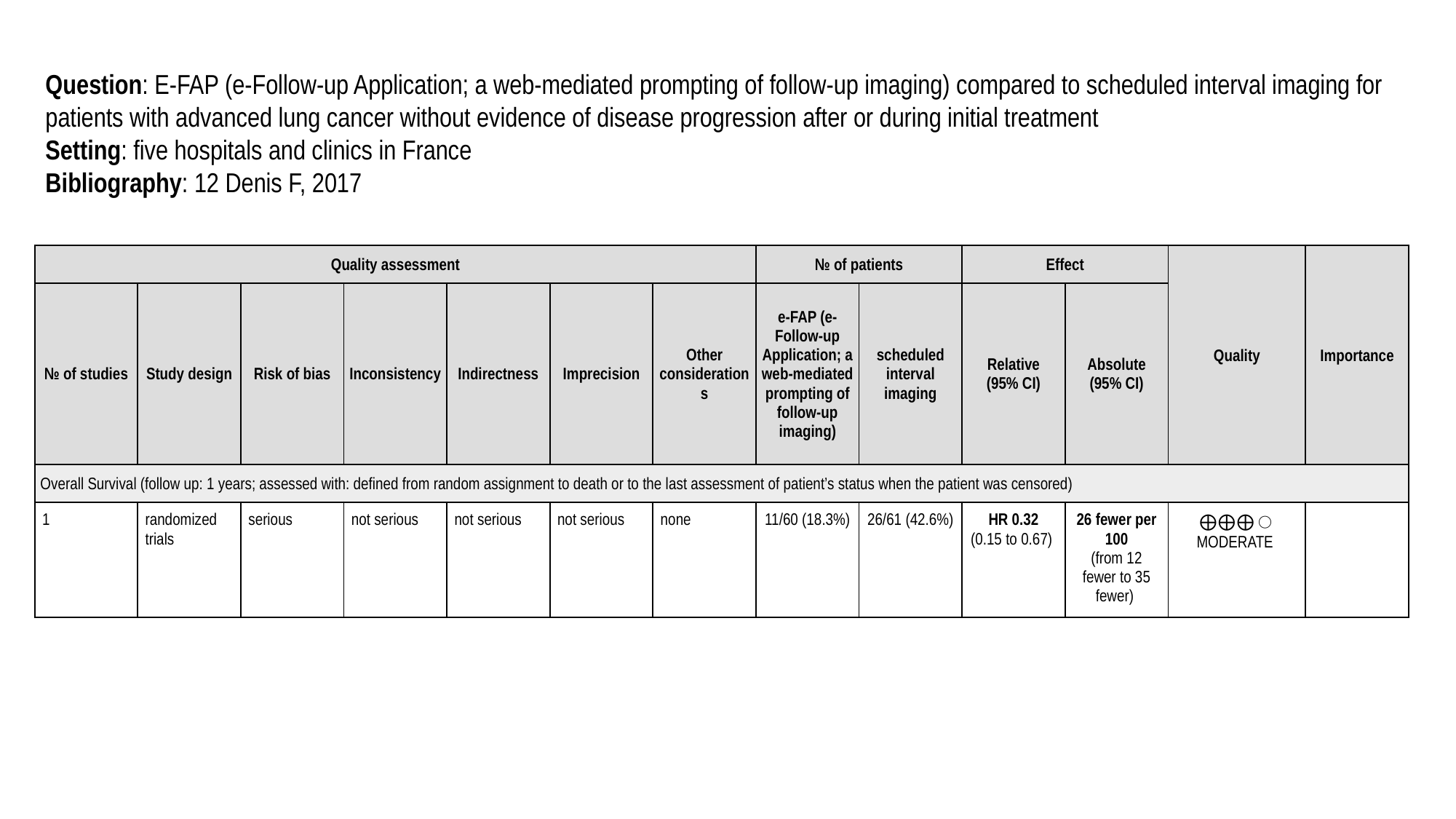

Question: E-FAP (e-Follow-up Application; a web-mediated prompting of follow-up imaging) compared to scheduled interval imaging for patients with advanced lung cancer without evidence of disease progression after or during initial treatment
Setting: five hospitals and clinics in France
Bibliography: 12 Denis F, 2017
| Quality assessment | | | | | | | № of patients | | Effect | | Quality | Importance |
| --- | --- | --- | --- | --- | --- | --- | --- | --- | --- | --- | --- | --- |
| № of studies | Study design | Risk of bias | Inconsistency | Indirectness | Imprecision | Other considerations | e-FAP (e-Follow-up Application; a web-mediated prompting of follow-up imaging) | scheduled interval imaging | Relative (95% CI) | Absolute (95% CI) | | |
| Overall Survival (follow up: 1 years; assessed with: defined from random assignment to death or to the last assessment of patient’s status when the patient was censored) | | | | | | | | | | | | |
| 1 | randomized trials | serious | not serious | not serious | not serious | none | 11/60 (18.3%) | 26/61 (42.6%) | HR 0.32 (0.15 to 0.67) | 26 fewer per 100 (from 12 fewer to 35 fewer) | ⨁⨁⨁◯ MODERATE | |

## Slide 13
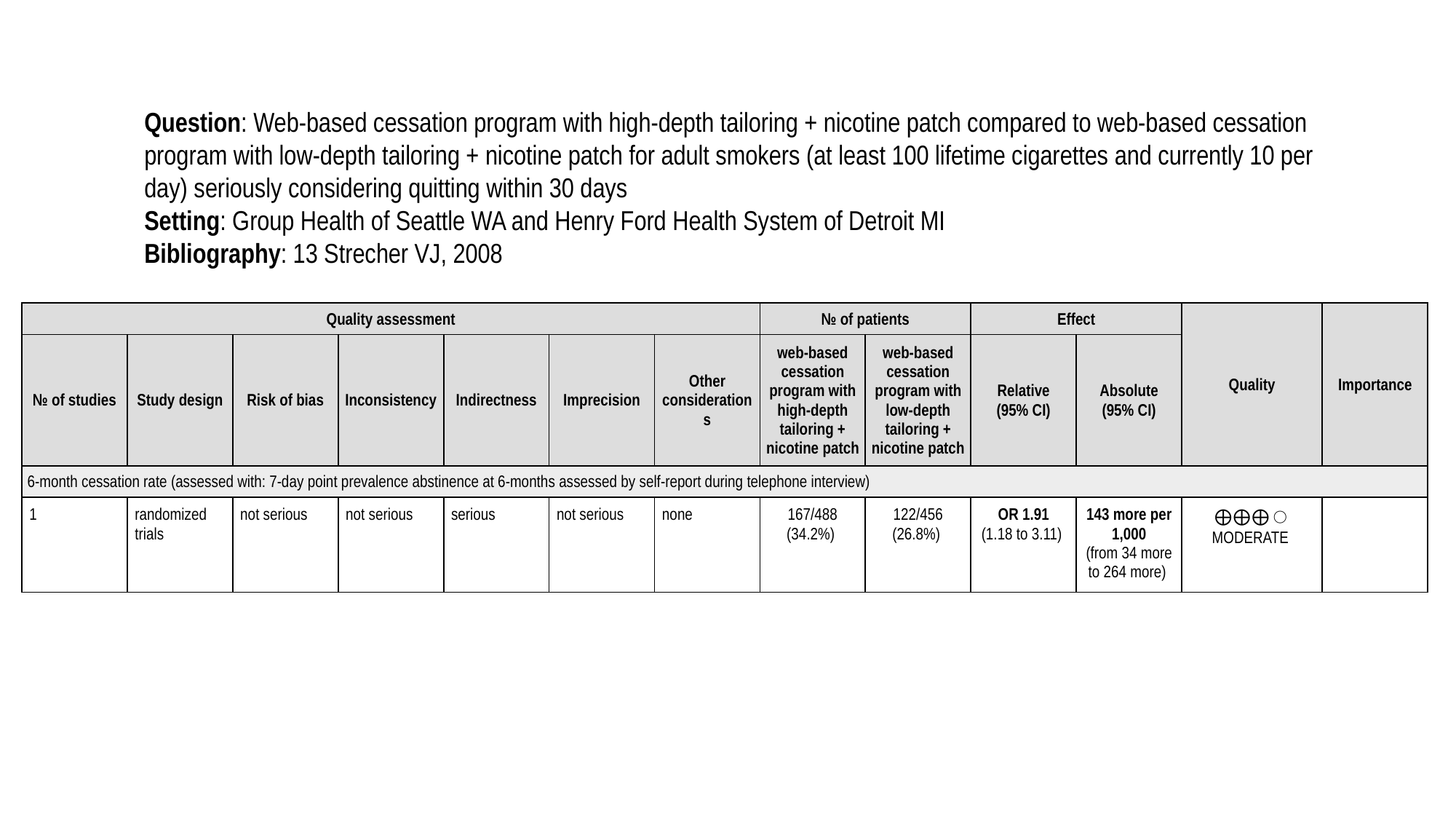

Question: Web-based cessation program with high-depth tailoring + nicotine patch compared to web-based cessation program with low-depth tailoring + nicotine patch for adult smokers (at least 100 lifetime cigarettes and currently 10 per day) seriously considering quitting within 30 days
Setting: Group Health of Seattle WA and Henry Ford Health System of Detroit MI
Bibliography: 13 Strecher VJ, 2008
| Quality assessment | | | | | | | № of patients | | Effect | | Quality | Importance |
| --- | --- | --- | --- | --- | --- | --- | --- | --- | --- | --- | --- | --- |
| № of studies | Study design | Risk of bias | Inconsistency | Indirectness | Imprecision | Other considerations | web-based cessation program with high-depth tailoring + nicotine patch | web-based cessation program with low-depth tailoring + nicotine patch | Relative (95% CI) | Absolute (95% CI) | | |
| 6-month cessation rate (assessed with: 7-day point prevalence abstinence at 6-months assessed by self-report during telephone interview) | | | | | | | | | | | | |
| 1 | randomized trials | not serious | not serious | serious | not serious | none | 167/488 (34.2%) | 122/456 (26.8%) | OR 1.91 (1.18 to 3.11) | 143 more per 1,000 (from 34 more to 264 more) | ⨁⨁⨁◯ MODERATE | |

## Slide 14
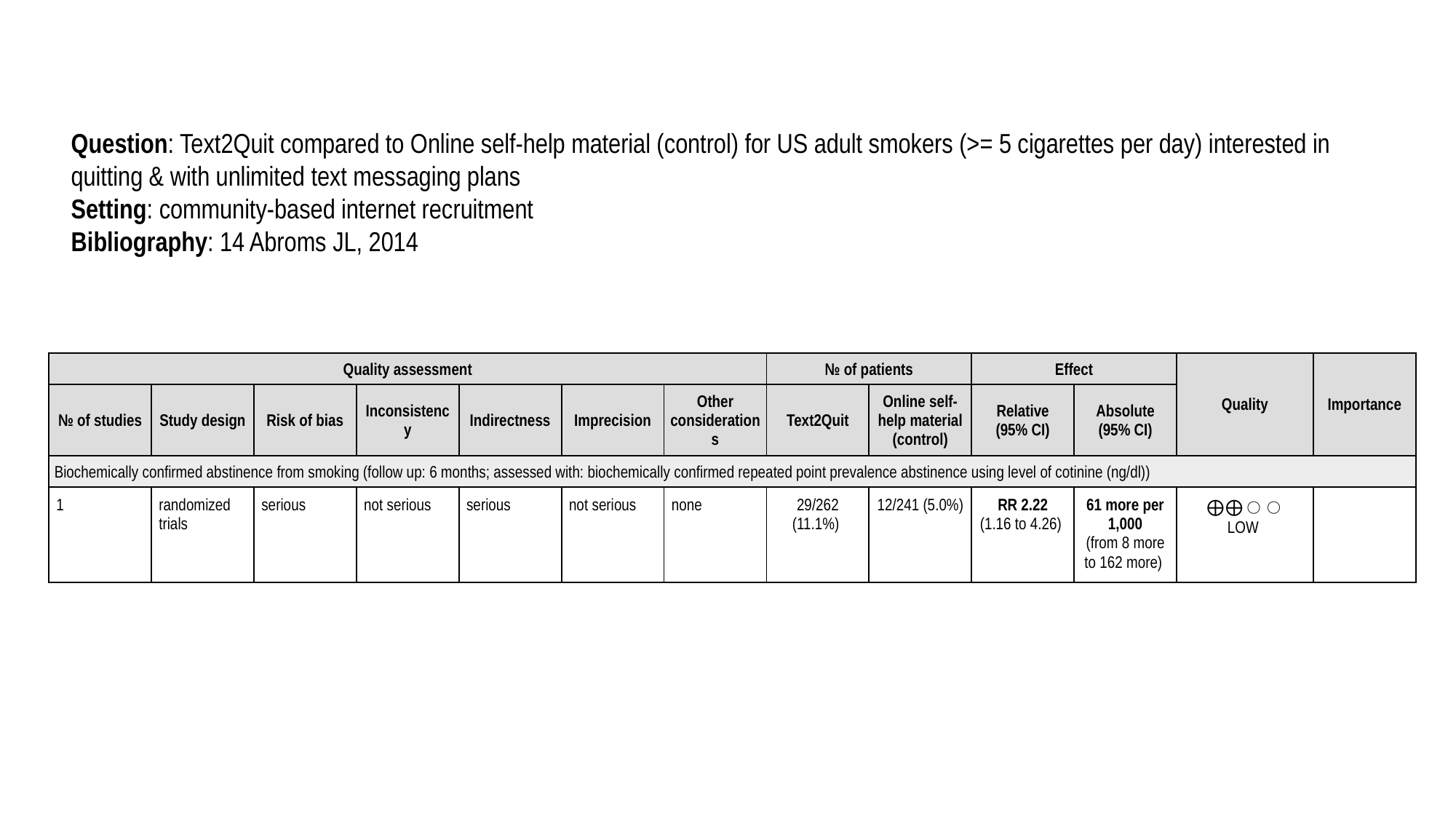

Question: Text2Quit compared to Online self-help material (control) for US adult smokers (>= 5 cigarettes per day) interested in quitting & with unlimited text messaging plans
Setting: community-based internet recruitment
Bibliography: 14 Abroms JL, 2014
| Quality assessment | | | | | | | № of patients | | Effect | | Quality | Importance |
| --- | --- | --- | --- | --- | --- | --- | --- | --- | --- | --- | --- | --- |
| № of studies | Study design | Risk of bias | Inconsistency | Indirectness | Imprecision | Other considerations | Text2Quit | Online self-help material (control) | Relative (95% CI) | Absolute (95% CI) | | |
| Biochemically confirmed abstinence from smoking (follow up: 6 months; assessed with: biochemically confirmed repeated point prevalence abstinence using level of cotinine (ng/dl)) | | | | | | | | | | | | |
| 1 | randomized trials | serious | not serious | serious | not serious | none | 29/262 (11.1%) | 12/241 (5.0%) | RR 2.22 (1.16 to 4.26) | 61 more per 1,000 (from 8 more to 162 more) | ⨁⨁◯◯ LOW | |
